# Microorganisms in subarctic soils are depleted of ribosomes under short-, medium-, and long-term warming

**DOI:** 10.1093/ismejo/wrae081

**Published:** 2024-05-09

**Authors:** Andrea Söllinger, Laureen S Ahlers, Mathilde Borg Dahl, Páll Sigurðsson, Coline Le Noir de Carlan, Biplabi Bhattarai, Christoph Gall, Victoria S Martin, Cornelia Rottensteiner, Liabo L Motleleng, Eva Marie Breines, Erik Verbruggen, Ivika Ostonen, Bjarni D Sigurdsson, Andreas Richter, Alexander T Tveit

**Affiliations:** Department of Arctic and Marine Biology, UiT The Arctic University of Norway, Framstredet 39, 9019 Tromsø, Norway; Department of Arctic and Marine Biology, UiT The Arctic University of Norway, Framstredet 39, 9019 Tromsø, Norway; Institute of Microbiology, University of Greifswald, Felix-Hausdorff-Straße 8, 17489 Greifswald, Germany; Faculty of Environmental and Forest Sciences, Agricultural University of Iceland, Árleynir 22, 112 Reykjavík, Iceland; Present address: Icelandic Forest Service, Austurvegi 3, 800 Selfoss, Iceland; Research Group Plants and Ecosystems (PLECO), University of Antwerp, Universiteitsplein 1, 2610 Wilrijk, Belgium; Department of Geography, University of Tartu, Vanemuise 46, 51003 Tartu, Estonia; Centre for Microbiology and Environmental Systems Science, University of Vienna, Djerassiplatz 1, 1030 Vienna, Austria; Centre for Microbiology and Environmental Systems Science, University of Vienna, Djerassiplatz 1, 1030 Vienna, Austria; Centre for Microbiology and Environmental Systems Science, University of Vienna, Djerassiplatz 1, 1030 Vienna, Austria; Department of Arctic and Marine Biology, UiT The Arctic University of Norway, Framstredet 39, 9019 Tromsø, Norway; Department of Arctic and Marine Biology, UiT The Arctic University of Norway, Framstredet 39, 9019 Tromsø, Norway; Research Group Plants and Ecosystems (PLECO), University of Antwerp, Universiteitsplein 1, 2610 Wilrijk, Belgium; Department of Geography, University of Tartu, Vanemuise 46, 51003 Tartu, Estonia; Faculty of Environmental and Forest Sciences, Agricultural University of Iceland, Árleynir 22, 112 Reykjavík, Iceland; Centre for Microbiology and Environmental Systems Science, University of Vienna, Djerassiplatz 1, 1030 Vienna, Austria; Department of Arctic and Marine Biology, UiT The Arctic University of Norway, Framstredet 39, 9019 Tromsø, Norway

**Keywords:** microbial physiology, temperature response, soil warming, seasonal temperature changes, protein biosynthesis, RNA, DNA, Iceland, grassland soil, forest soil

## Abstract

Physiological responses of soil microorganisms to global warming are important for soil ecosystem function and the terrestrial carbon cycle. Here, we investigate the effects of weeks, years, and decades of soil warming across seasons and time on the microbial protein biosynthesis machineries (i.e. ribosomes), the most abundant cellular macromolecular complexes, using RNA:DNA and RNA:MBC (microbial biomass carbon) ratios as proxies for cellular ribosome contents. We compared warmed soils and non-warmed controls of 15 replicated subarctic grassland and forest soil temperature gradients subject to natural geothermal warming. RNA:DNA ratios tended to be lower in the warmed soils during summer and autumn, independent of warming duration (6 weeks, 8–14 years, and > 50 years), warming intensity (+3°C, +6°C, and +9°C), and ecosystem type. With increasing temperatures, RNA:MBC ratios were also decreasing. Additionally, seasonal RNA:DNA ratios of the consecutively sampled forest showed the same temperature-driven pattern. This suggests that subarctic soil microorganisms are depleted of ribosomes under warm conditions and the lack of consistent relationships with other physicochemical parameters besides temperature further suggests temperature as key driver. Furthermore, in incubation experiments, we measured significantly higher CO_2_ emission rates per unit of RNA from short- and long-term warmed soils compared to non-warmed controls. In conclusion, ribosome reduction may represent a widespread microbial physiological response to warming that offers a selective advantage at higher temperatures, as energy and matter can be reallocated from ribosome synthesis to other processes including substrate uptake and turnover. This way, ribosome reduction could have a substantial effect on soil carbon dynamics.

## Introduction

The complex microbial communities residing in soils are responsible for ~50% of the carbon (C) efflux from terrestrial ecosystems by releasing carbon dioxide (CO_2_), an end-product of the microbial oxidation of soil organic carbon (SOC), to the atmosphere [1]. However, microorganisms themselves contribute living and dead biomass to the SOC pool [2] and the balance between C input, soil microbial respiration, and SOC stabilization will be decisive for whether soils will act as atmospheric C sources or sinks in a warmer future. Microbial responses to global warming may impact this balance substantially, and it has been proposed that projections of soil–CO_2_–climate feedbacks can be considerably improved by integrating microbial processes and underlying microbial physiologies [3, 4].

Microbial responses to long-term soil warming range from (i) structural changes in the community composition (e.g. [5, 6]), to (ii) quantitative changes, such as an overall decrease in fungal and microbial biomass (e.g. [7, 8]), and (iii) functional and physiological changes, including shifts in extracellular enzyme pools and activities (e.g. [9–11]) as well as altered growth rates (e.g. [12–14]). However, general soil microbial responses to global warming and the consequences for C cycling across soil types, climate zones, and time may not be easily inferred from these individual observations because of the large variability in net primary productivity and belowground carbon allocation, water and nutrient availability, soil physics and chemistry, microbial community composition, and evolutionary history between studied soil ecosystems [15]. Furthermore, it has been shown that seasonal microbial community dynamics can be restructured by warming, e.g. by decreasing the relative importance of stochastic processes in microbial community assembly in winter but increasing it in summer [16]. This highlights the need for integrating seasonality and changing conditions over time when studying microbial responses to warming and related consequences for terrestrial C cycling.

Recently, we proposed that grassland soil microorganisms exposed to warming reduce their ribosome contents and that this downregulation of the cellular protein biosynthesis machinery is facilitated by accelerated protein biosynthesis rates per ribosome at higher temperatures [17]. Consequentially, warming-induced ribosome reduction allows energy and matter to be reallocated to alternative functions and microbial processes. We further suggested that soil warming is not only increasing microbial activities directly via increased metabolic reaction rates, but possibly also indirectly via ribosome reduction and altered resource reallocation.

The goal of this study was to explore the temporal dynamics of temperature-driven regulation of the ribosome content of soil microbial communities. We compared RNA contents per unit of DNA and per unit of microbial biomass C (MBC), as two proxies for microbial cellular ribosome contents, of non-warmed and short-, medium-, and long-term warmed subarctic grassland and forest soils (+3°C, +6°C, and + 9°C of natural geothermal warming for years and decades and + 6°C of warming soil microcosms for weeks). Soils were sampled over the course of 4 years to determine whether ribosome reduction upon soil warming is consistent across changing conditions associated to different seasons, warming intensities, time, and soil ecosystems. To reveal potential drivers of ribosome reduction, we further assessed possible relationships with a range of physicochemical soil parameters and biological characteristics, including soil temperature, gravimetric water content (GWC), pH, total soil C and nitrogen (N) contents, dissolved organic C (DOC), total dissolved N (TDN), MBC and microbial biomass N (MBN) contents, microbial RNA and DNA contents, fine root biomass, and soil microbial respiration.

## Materials and methods

### Sites and sampling

**Figure 1:**
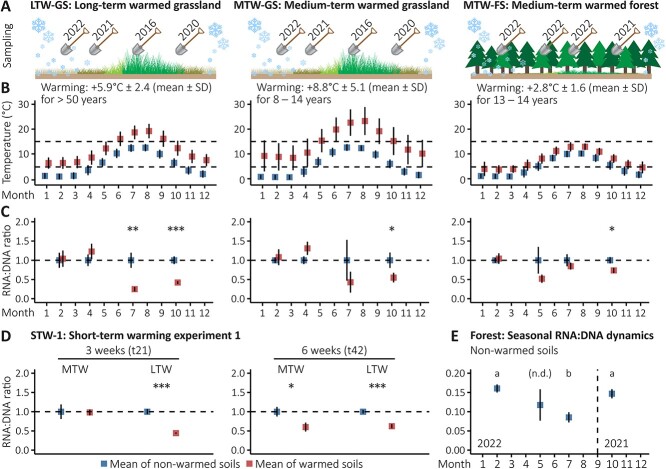
RNA:DNA ratios. (A) Overview of the seasonal soil sampling of the LTW-GS, the MTW-GS, and the MTW-FSs. (B) Mean soil temperatures per month (error bars = SD) measured at 10 cm soil depth of non-warmed control soils (*n* = 5 per site, blue) and warmed soils (*n* = 5 per site, red) sampled; dashed lines: + 5°C and + 15°C. (C) Mean fold-change of RNA:DNA ratios between non-warmed (blue) and warmed soils (red) per site (LTW-GS, MTW-GS, and MTW-FS) and sampled month; calculated by setting the mean of non-warmed control soils of each timepoint to one (dashed line). Values above the dashed line indicate an increase in cellular ribosome contents, whereas values below indicate a decrease. Hypothesizing that RNA:DNA ratios are lower in warmed soils, one-sided *t* tests were performed to test for significant differences (C, D); ^*^P < .05, ^*^^*^P < .01, ^*^^*^^*^P < .001; see Supplementary Tables S6 and S7 for details and exact *P* values. (D) Mean fold-change of RNA:DNA ratios between non-warmed controls (blue, incubated at 7°C) and short-term warmed grassland soils (red, incubated at 13°C); *n* = 5 per site and timepoint, see Supplementary Fig. S2A for details on the experimental setup. (E) Seasonal RNA:DNA dynamics (absolute ratios) of the consecutively sampled non-warmed forest soils. The lowest RNA:DNA ratios were observed at the highest temperatures in summer, albeit significantly lower only if the may ratios are excluded from pairwise *t* tests or *P* values are not corrected for multiple testing (see Supplementary Table S8 for details and exact *P* values). Error bars (C–E) represent standard error of the mean.

We sampled 15 natural soil temperature gradients of the Icelandic ForHot Experiment [18] (Supplementary Fig. S1), that are powered by geothermal activity, over the course of several years and seasons, i.e. in July 2016, October 2020, April 2021, October 2021, February 2022, May 2022, and July 2022 (Fig. 1A, Supplementary Table S1). The 15 gradients are located at three different sites (*n* = 5 replicated gradients per site), a long-term warmed grassland site (LTW-GS) that has been warmed for >50 years, a medium-term warmed grassland site (MTW-GS) that emerged nearby after an earthquake in 2008, and a medium-term warmed forest site (MTW-FS) next to MTW-GS that also emerged after the earthquake. The soils of all three sites are classified as Silandic Andosols and the vegetational covers of the grasslands are dominated by *Agrostis capillaris*, whereas the forest is dominated by *Picea sitchensis*, with no significant understorey vegetation (see [18] for more details on the ForHot sites, their establishment, and their suitability as natural climate change experiment). All gradients include non-warmed control plots (A_T_) and warmed plots with an anticipated temperature increase of +6°C (E_T_); between A_T_ and E_T_ plots are three more plots, B_T_, C_T_, and D_T_, with intermediate warming intensities (Supplementary Fig. S1). On all plots, soil temperatures at 10 cm depth have been hourly logged since 2013, showing stable levels of soil warming throughout seasons (Fig. 1B), but also revealing a significantly higher mean hourly temperature difference between A_T_ and E_T_ plots at MTW-GS (+8.8°C) compared to LTW-GS (+5.9°C) (two-sided *t* test, *n* = 639 974, *t* = 283.41, *P* < 2.2 × 10^−16^). The E_T_ plots at MTW-FS are partly damaged due to dying and falling trees from adjacent areas with elevated warming (> +6°C of warming). Selecting E_T_ and A_T_ plots was pragmatical because a much broader range of context data was available for the grassland E_T_ and A_T_ plots than other plots. To avoid dealing with the interference of dying trees on the temperature effect, the MTW-FS-E_T_ plots were replaced by D_T_ plots, which showed a mean hourly temperature difference to the non-warmed control plots of +2.8°C (Fig. 1B). Hence, we compared non-warmed soils of the three sites (MTW-FS-A_T_, LTW-GS-A_T_, MTW-GS-A_T_) with +3°C (MTW-FS-D_T_), +6°C (LTW-GS-E_T_), and +9°C (MTW-GS-E_T_) warmed soils, respectively.

### Biological and physicochemical parameters

Total DNA and RNA contents of non-warmed control soils (A_T_) and warmed soils (D_T_ and E_T_) were obtained by extracting total nucleic acids from flash-frozen soil samples (soil cores taken from the upper 10 cm, ground in liquid N and homogenized) using a quantitative phenol–chloroform extraction protocol to allow a comparison within and between seasons and sites; see [17, 19] and Supplementary Table S2. Total RNA and DNA contents were quantified using a Qubit 2.0 Fluorometer (Thermo Fisher Scientific, Waltham, MA, USA) and the Qubit RNA HS Assay Kit and the Qubit dsDNA HS Assay Kit, respectively. Physicochemical soil parameters and microbial biomass C (MBC) and microbial biomass N (MBN) contents were obtained as described [17] using standard protocols and procedures. Briefly, total C and N contents were analysed in dried soil aliquots using an elemental analyser coupled to an isotope ratio mass spectrometer (EA-IRMS; EA1110 coupled via a ConFlo III interface to a DeltaPLUS IRMS, Thermo Fisher Scientific). Total DOC and TDN concentrations were obtained in KCl extracts (in 1:5 dilutions with water) on a DOC/TDN analyser (Shimadzu TOC-VCPH/CPNTNM-1 analyser, Kyoto, Japan), after extracting 2 g fresh soil aliquots with 15 ml of a 1 M KCl solution for 30 min at room temperature. MBC and MBN contents were determined via a chloroform–fumigation extraction method after [20] (48 h incubation period of 2 g fresh soil aliquots followed by KCl extraction, as described above) and calculated as the difference between fumigated samples and nonfumigated controls. Thus, the presented MBC and MBN contents represent the extractable fraction of the total MBC and MBN. Fine root biomass (mg dry roots g^−1^ dry weight (DW) soil) was obtained from a second set of soil cores sampled in parallel and next to the soil cores used for total nucleic acid extractions in October 2020 and April 2021. After being freeze-dried, soil-free fine roots (< 0.5 mm) were collected in a timed and standardized way across samples.

### Incubation experiments

Fresh LTW-GS and MTW-GS samples, taken in October 2020 and October 2021, were used in two complementary short-term warming incubation experiments (STW-1 and STW-2, first and second short-term warming experiment, respectively), directly conducted upon the return from the respective sampling campaigns (see Supplementary Fig. S2 for details on the experimental setup). Prior to the start of the experiments, the soils were sieved (2 mm mesh size) and pre-incubated in microcosm bottles for 3 weeks at their respective *in situ* temperature (i.e. 7°C, the approximate mean *in situ* temperature of the A_T_ grassland soils in October; or 13°C, the approximate mean *in situ* temperature of the E_T_ grassland soils in October), to allow the soils to equilibrate. All microcosms were incubated in the dark throughout the preincubations and the main experiments.

#### *First short-term warming experiment* (STW-1)

Sieved grassland soils from two gradients, MTW-1-A_T_ and LTW-4-A_T_, were separated into ten 100 ml serum bottles per gradient, each containing ~22 g of soil (Supplementary Fig. S2A). After the preincubation period, the 10 replicates of each soil were split. Five replicates were further incubated at 7°C (control incubations), whereas the other five replicates were incubated at 13°C (+6°C of warming). All soil microcosm bottles were incubated for 6 weeks. The rational for limiting the short-term warming to a maximum of 6 weeks was based on a previous short-term warming incubation experiment with LTW-GS soils [12], in which a soil C response was observed after 6 weeks; i.e. DOC showed a negative response although total soil C was not yet significantly reduced. Subsamples (~1.5 g) for molecular analyses were taken at the beginning of the experiment (t0, right before bottling, “timepoint 0”), after 3 weeks of incubation (t21) and at the end of the incubation (t42), flash frozen, ground in liquid nitrogen, and stored at −80°C until total nucleic acids were extracted as described above. GWC was determined at t0 and t42 by drying 2 g of soil (24 h at 100°C). GWC of t21 was estimated assuming a linear decrease (i.e. drying) over time. Total C and N, total DOC and N (TDN), and MBC and MBN contents were analysed as described above. Sieved soil from MTW-1-E_T_ and LTW-4-E_T_, respectively, separated into five 100 ml serum bottles, each containing ~22 g of soil, acted as LTW controls (Supplementary Fig. S2A). Subsamples for molecular analysis, GWC, and total and MBC and MBN were taken after 3 weeks of incubation (i.e. the preincubation time) at the approximated mean *in situ* long-term warming October soil temperature of 13°C and processed as described above.

#### Second short-term warming experiment (STW-2)

Sieved soils from A_T_ and E_T_ plots of all five replicated long-term warmed grassland soil temperature gradients were bottled (Supplementary Fig. S2B). The five A_T_ soils were each separated into two 500 ml glass bottles, containing ~100 g of soil, and one bottle per E_T_ soil was prepared, containing also ~100 g of soil. The resulting 15 bottles were pre-incubated for 3 weeks at their respective approximated mean *in situ* October soil temperature, i.e. 7°C (A_T_ soil bottles) and 13°C (E_T_ soil bottles). After the preincubation, one set of A_T_ microcosms was exposed to +6°C of warming, resulting in an incubation temperature of 13°C (“Short-term warming at E_T_”), the other set of A_T_ microcosms was kept at 7°C (“Non-warmed control at A_T_”), and the E_T_ microcosms were kept at 13°C (“Long-term warming control at E_T_”); Supplementary Fig. S2B. All soil microcosm bottles were incubated for 3 weeks. Subsamples (~1.5 g) for molecular analysis were taken at the beginning of the experiment (t0) and after 3 weeks of incubation (t21), flash frozen, ground in liquid nitrogen, and stored at −80°C until total nucleic acids were extracted as described above. GWC was determined at t0 and t21 by drying 2 g of soil (24 h at 100°C). Soil pH at t0 and t21 was measured at room temperature using 2 g of fresh soil suspended in 5 ml of a 0.05 M CaCl_2_ solution. Total C and N, total DOC and TDN, and MBC and MBN contents were analysed as described above.

#### Gas chromatography

Soil CO_2_ emission rates (nM CO_2_ h^−1^ g^−1^ DW soil) (i.e. soil microbial respiration rates) of both STW experiments were obtained by measuring 24 h CO_2_ accumulations in the headspace of the microcosm bottles regularly throughout the experiments using a gas chromatograph (SRI 8610C, SRI Instruments, Bad Honnef, Germany; equipped with a flame ionising detector) and inferring CO_2_ concentrations via standard curves created from gases with known CO_2_ concentrations applying the general gas equation. During the 24-h periods, the bottles were sealed with air-tight rubber stoppers; between measurements, the bottles were aerated and closed with aluminium foil (same as during the preincubation).

### Metatranscriptomics

A total of 16 soil metatranscriptomes, originating from the LTW and the MTW grassland sites sampled in July 2016 [6, 11, 17], were analysed to test for significant differences in relative community-level rRNA operon copy numbers between non-warmed control soils (A_T_) and warmed soils (E_T_). Subsamples of 200 000 SSU rRNA reads have been taxonomically classified previously using CREST3 [21] and a lowest common ancestor approach [6]. Bacterial reads, which accounted for >99% of all prokaryotic reads, were extracted and further analysed. Mean rRNA operon copy numbers were obtained from the ribosomal RNA operon database (rrnDB) v5.8 (ref. [22]). For each bacterial read, the lowest assigned taxonomic level with a match in the rrnDB was selected, and copy-number corrected relative abundances were calculated by dividing relative rRNA read abundances of bacterial taxa by their mean rRNA operon copy numbers. Two-sided *t*-tests were used to test for significant differences in copy-number corrected relative abundances between LTW-A_T_ and LTW-E_T_ as well as MTW-A_T_ and MTW-E_T_. Whereas higher copy number corrected relative abundances are indicative for lower relative community-level rRNA operon copy numbers. Subsequently, we estimated community mean rRNA operon copy numbers and tested for significant differences between LTW-A_T_ and LTW-E_T_ as well as MTW-A_T_ and MTW-E_T_. Furthermore, the functionally annotated mRNA reads, assigned to metabolic pathways and functional complexes defined in the Kyoto Encyclopedia of Genes and Genomes (KEGG) Orthology database, of the 16 soil metatranscriptomes [17] were re-analysed to investigate transcriptional investment in carbohydrate metabolisms and ribosomes.

### Statistical analysis

We used Rstudio (rstudio.com) and R (r-project.org), version 4.2.2 to analyse the data, perform statistical tests, and graphically display the results. Hypothesizing that RNA:DNA ratios are lower in warmed soils, one-sided *t*-tests (using the basic R function *t.test*) were performed to test for significant differences between non-warmed control soils and short-, medium- and long-term warmed soils at each timepoint of the seasonal survey and the short-term warming experiments. Pairwise *t*-tests (using the basic R function *pairwise.t.test*) were used to test for significant differences in RNA:DNA ratios of the consecutively sampled non-warmed forest soils. The Benjamini–Hochberg procedure (p.adjust.method = “BH”) was used to correct *P* values for multiple testing. Correlations between biological and physicochemical soil parameters were investigated using the basic R function *cor.test* (method = “spearman”). Two-sided *t* tests (*t.test*) were performed to test for significant differences in CO_2_ emission rates from non-warmed, long-term, and short-term warmed soil incubations at different timepoints, as well as significant differences in water contents, substrate availabilities, and fine root biomass between non-warmed and warmed soils. Pairwise *t* tests (using the basic R function *pairwise.t.test*) were used to test for significant differences in fine root biomass between seasons. The Benjamini–Hochberg procedure (p.adjust.method = “BH”) was used to correct *P* values for multiple testing. Multiple linear regression models (using the basic R function *lm*) were used to test the effect of multiple distinct predictor variables on RNA:MBC and RNA:DNA ratios. Pairwise interactive effects of temperature and other environmental variables were evaluated by integrating interactive effects in analysis of variance (ANOVA) models (using the basic R function *aov*). Two-sided *t* tests (*t.test*) were employed to test for significant differences in copy-number corrected relative abundances of bacterial taxa, estimated community mean rRNA operon copy numbers, and relative transcriptional investments of the soil microbial communities between non-warmed and warmed soils.

See Supplementary Information, Supplementary Figs. S1**–**S3, and Supplementary Tables S1–S5 for more details on sampling sites, sample processing, short-term warming experiments, and data analysis using R [23]. The raw sequence data are available at the NCBI Sequence Read Archive (SRA); BioProject ID: PRJNA663238, accession numbers SAMN16124403–SAMN16124422. All underlying data and scripts needed to replicate the presented analyses are available on DataverseNO (10.18710/OW27B6).

## Results and discussion

### RNA:DNA and RNA:MBC ratios indicate ribosome reduction in response to warming

Throughout the growing season, *in situ* RNA:DNA ratios at the long- and medium-term warmed grassland and forest sites (Fig. 1A and B) showed an overall trend towards lower RNA:DNA ratios in warmed soils compared to their non-warmed counterparts (Fig. 1C). Significantly lower RNA:DNA ratios were observed in the warmed soils in summer (LTW-GS) and in autumn (LTW-GS, MTW-GS, and MTW-FS) but not in winter and late winter/early spring. Likewise, 6 weeks of warming led to significantly lower RNA:DNA ratios in the grassland soils originating from non-warmed plots of both the MTW and the LTW grassland sites, and for the latter, this result was already observed after 3 weeks of warming in the first short-term warming experiment but not in the second (Fig. 1D, Supplementary Fig. S4). Taken together, this indicates that microbial cells consistently respond to weeks (>3), years, and decades of warming by reducing their ribosome contents. In autumn, a significant reduction in RNA:DNA ratios in warmed soils compared to their non-warmed counterparts could be observed at all three sites *in situ* and in our 6-week warming experiment conducted with autumn soils (Fig. 1C and D). This suggests that autumn conditions, potentially including autumn temperature ranges and plant senescence, promote ribosome reduction upon warming. Furthermore, the absence of RNA:DNA ratio reduction in the warmed soils during winter suggests the existence of a seasonal dynamic and perhaps an absolute temperature threshold for triggering this physiological response. The RNA:DNA ratios of the consecutively sampled forest soils highlight this seasonal dynamic, being highest in winter at the lowest temperatures and lowest in summer at the highest temperatures (Fig. 1E). Higher protein biosynthesis rates per ribosome at higher temperatures [24] might facilitate ribosome reduction during summer, whereas the buildup of ribosomes during winter might be aided by an increased half-life time of recently produced RNA (4 days at 20°C vs. 15.8 days at 4°C) [25].

**Figure 2:**
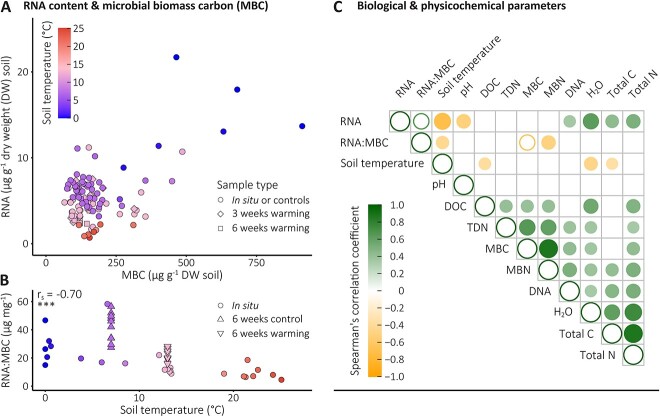
Relationship between RNA content and microbial biomass carbon and correlations between biological and physicochemical parameters. (A) RNA and MBC contents at soil temperatures ranging from 0.0 to 25.1°C; all *in situ* grassland soil samples and samples from the short-term warming experiments with measured (*n* = 85) and extrapolated MBC contents (*n* = 30) are included (see Supplementary Information section “Biological and physicochemical soil properties” for details on the MBC extrapolation). “*In situ* or controls” represent *in situ* samples taken from the medium- and long-term warmed grassland soils and their non-warmed counterparts as well as samples taken before the start of the short-term warming (at t0) and controls of the short-term warming experiments (Supplementary Fig. S2). “3 weeks warming” and “6 weeks warming” represent samples taken after three and 6 weeks of experimental warming, respectively. (B) RNA:MBC ratios of all *in situ* samples and after 6 weeks of incubation (*n* = 46). Spearman’s rank correlation coefficient (r_s_) is given in the left corner (*P* = 4.9 × 10^−8^). (C) Correlations between biological and physicochemical soil parameters (same dataset as in (A)). Only significant correlations with a *P* < .001 are shown; size and colour of the dots indicate the strength and direction of the correlation (green, positive correlation; yellow, negative correlation; hollow dots, not applicable). See Supplementary Table S9 for more details, including all significant correlations, units, and exact *P* values.

The RNA:DNA ratios observed in our soils represent proxies for microbial cellular ribosome contents that potentially can be biased by varying amounts of extracellular DNA derived from microbial necromass [26]. Therefore, we employed RNA contents per unit of microbial biomass carbon (MBC) as a second proxy for microbial cellular ribosome contents. In the following, we describe and discuss observations made on a subset of *in situ* grassland soil samples (July 2016, February 2022; *n* = 26) and samples from the short-term warming experiments (*n* = 89), for which we have quantified MBC contents (total *n* = 115; Supplementary Tables S3 and S5). We observed the highest RNA contents per unit of MBC at temperatures below 10°C and the lowest RNA contents per unit of MBC at temperatures above 20°C (Fig. 2A and B), suggesting ribosome depletion at higher temperatures. This matched the observed RNA contents per unit of microbial N and the RNA contents per unit of DNA (Supplementary Fig. S5). MBC and MBN contents (mg g^−1^ DW soil) from these grassland samples (*n* = 115) showed very weak and insignificant correlations with soil temperatures (*r*_s_ = −0.11 and − 0.16, respectively, *P* > .05), whereas RNA contents (μg g^−1^ DW soil) showed a strong negative correlation with soil temperatures (*r*_s_ = −0.70, *P* < 2.2 × 10^−16^) (Fig. 2C, Supplementary Table S9), resulting in the observed pattern of decreasing RNA contents per unit of MBC with increasing temperatures (Fig. 2A and B). However, RNA contents (μg g^−1^ DW soil) showed a nearly as strong positive correlation with soil water contents (GWC; *n* = 115, *r*_s_ = 0.63, *P* = 3.6 × 10^−14^) (Fig. 2C, Supplementary Table S9). Accordingly, the mapping of soil water contents onto the RNA content per unit of MBC (Supplementary Fig. S6A) revealed an inverted pattern compared to the mapping of temperature (Fig. 2A), with ribosome-depleted biomass at lower water contents. In contrast, the mapping of pH, DOC, TDN, total C, and total N contents onto the RNA content per unit of MBC showed less pronounced patterns (Supplementary Fig. S6B–F), and no or weak to moderate correlations (r_s_ > −0.5 and < 0.5) with RNA contents and RNA:MBC ratios were observed (Fig. 2C, Supplementary Table S9).

### Effects of soil water and soil C and N contents on RNA:DNA and RNA:MBC ratios

We wanted to investigate further the strong correlation between RNA content and water content seen in the subset of grassland soil samples for which MBC measurements were available. Revisiting *in situ* grassland and forest soil water contents across all seasons, which ranged from 27 to 70% (mean = 48% ± 10% SD; total n = 112), revealed that significantly lower RNA:DNA ratios in warmed soils were not consistently co-occurring with significantly lower water contents (Supplementary Fig. S7A, Supplementary Table S10). *In situ* RNA contents (μg g^−1^ DW soil) showed a positive correlation with *in situ* soil water contents in the grasslands (LTW-GS: *n* = 34, *r*_s_ = 0.70, *P* = 7.1 × 10^−6^; MTW-GS: *n* = 38, *r*_s_ = 0.62, *P* = .0001), whereas we observed a negative relationship between RNA and soil water contents in the forest samples (MTW-FS: *n* = 39, *r*_s_ = −0.38, *P* = .02; see Supplementary Table S10B). Accordingly, the forest soils showed the lowest RNA:DNA ratios in summer (Fig. 1E) when the water contents were the highest (mean = 58% ± 7% SD) and the highest RNA:DNA ratios in winter (Fig. 1E) when the water contents were the lowest (mean = 47% ± 5% SD). Furthermore, in the short-term warming experiments, RNA:DNA ratios were reduced by ~40% in the warmed soils compared to their non-warmed counterparts after 6 weeks (Fig. 1D), but water contents did not differ between warmed and non-warmed soils (Supplementary Fig. S7B).

Recently, it was suggested that substrate availability in long-term warmed soils and its seasonality indirectly affects microbial physiologies and by that controls ecosystem-scaled C cycling processes and seasonal dynamics [27]. However, we did not find substrate-related parameters (total and dissolved C and N contents) to consistently match the correlating changes in temperature and ribosome content proxies (Supplementary Figs S8 and S9). For example, in summer, the LTW grassland soils showed significantly lower RNA:DNA and RNA:MBC ratios compared to their non-warmed counterparts, whereas no significant differences in GWCs, total C, DOC, total N, and TDN contents were observed (Supplementary Fig. S8, Supplementary Table S11). In contrast, in winter, when RNA:DNA and RNA:MBC ratios did not differ significantly between warmed and non-warmed LTW grassland soils, soil water, and DOC contents were significantly lower in the warmed soils (*P* < .05) and total C and N showed a trend towards lower contents in the warmed soils (*P* < .08). Total C, DOC, total N, and TDN contents in the LTW grassland soils and their non-warmed counterparts were on average 1.4, 3.4, 1.2, and 15 times higher in winter compared to summer (Supplementary Table S12). However, even though total RNA contents (μg g^−1^ DW soil) were also highest in winter, in both the warmed and the non-warmed LTW-GS soils (Supplementary Fig. S9C), we did not see indications that total RNA contents in summer were limited by substrate quantities (Supplementary Fig. S9D). In fact, we observed an overall trend towards higher *substrate:RNA ratios* (i.e. total C:RNA, DOC:RNA, total N:RNA, and TDN:RNA ratios) in warmed soils compared to their non-warmed counterparts, especially in summer but also in short-term warmed soils (i.e. *P* ≤ .09 in seven out of eight tested ratios (LTW summer + short-term warming); see Supplementary Fig. S9D and Supplementary Table S13). Similarly, we did not observe a clear relationship between fine root biomass (mg dry roots g^−1^ DW soil) and seasonal temperature dependent differences in RNA:DNA ratios (Supplementary Fig. S10**,**Supplementary Table S14). The patterns of fine root biomass and RNA:DNA ratios were similar, i.e. showing significantly lower values in warmed soils compared to their non-warmed counterparts in October 2020 (a pattern also reported in October 2019 (ref. [28]), but not in April 2021. However fine root biomass, despite showing seasonal fluctuations, did neither differ significantly between seasons in the warmed nor the non-warmed soils (Supplementary Fig. S10). Nevertheless, microbial cellular ribosome contents may be influenced by qualitative and quantitative differences in root exudates [29] across seasons.

The strong relationship between temperature and microbial ribosome content proxies and the inconsistent relationships between microbial ribosome content proxies and other measured environmental variables, including soil water and soil C and N contents, suggests temperature as key driver of cellular ribosome content adjustments. In line with that, multiple linear regression models, applied to test the effect of multiple distinct predictor variables on RNA:MBC and RNA:DNA ratios, identified soil temperature as the sole consistently significant contributor (Supplementary Table S15), independent of the analysed dataset (i.e. the *in situ* dataset, the incubation experiment dataset, and the combined dataset including all *in situ* samples and incubation timepoints with significant differences in ribosome content proxies between warmed and non-warmed soils). Additionally, TDN content showed a significant effect on RNA:MBC and RNA:DNA ratios in the incubation experiment dataset and on RNA:DNA ratios in the combined dataset, whereas total C and total N contents showed a significant effect on RNA:MBC ratios in the *in situ* dataset (Supplementary Table S15). Testing pairwise interactive effects of temperature and other measured environmental variables on microbial ribosome content proxies did not reveal consistently significant interactive effects across the *in situ* dataset, the incubation experiment dataset, and the combined dataset (Supplementary Table S16). However, isolated significant interactive effects, such as a significant interactive effect of temperature and TDN on RNA:MBC ratios in the incubation experiment dataset and a significant interactive effect of temperature and soil water content on RNA:DNA ratios in the *in situ* dataset, were observed. Taking together, besides soil temperature, TDN and soil water content appear to be other important environmental variables, at times, influencing RNA:MBC and RNA:DNA ratios. Like with temperature, we observed a negative relationship between TDN content and both ribosome content proxies (Supplementary Table S15). However, as ribosomes and ribosomal proteins are generally N rich [30], it is seems counterintuitive that increased TDN contents are leading to ribosome reduction in microbial cells. It is more plausible that either the quality and quantity of bioavailable N in the TDN pool changed in the warmed soils or the N uptake capacity of the microbial communities decreased, leading to higher TDN contents. The counterintuitive relationship between TDN content and ribosome content proxies also highlights the need for further studies designed to specifically target the effects of a broader range of soil C, N, and also phosphorus (P) compounds on the cellular ribosome contents of soil microorganisms exposed to short-, medium-, long-term warming. For example, under stable temperature conditions, it has been shown that *Escherichia coli* uses different strategies to maintain the same protein production rates under C-, N- and P-limitation. Under C-limitation, inactive ribosomes that were not bound to mRNA, accumulated, whereas under N-limitation, elongation was slowed down, and only under P-limitation ribosome contents were reduced [31].

### Ribosome reduction: a physiological adjustment with potential consequences for C cycling

Changes in microbial community composition, such as the observation of increased relative and absolute abundances of bacteria with low ribosomal RNA (rRNA) operon copy numbers in warmed hardwood forest soils [5], could also influence RNA:DNA and RNA:MBC ratios. For example, multiple copies may allow faster ribosome synthesis under favourable conditions [32]. However, a recently published amplicon-based study investigating changes in bacterial community composition 4 years after the onset of warming in the medium-term warmed ForHot forest and grassland sites, revealed significant differences in bacterial community profiles only at warming intensities above +6°C relative to non-warmed conditions [33]. In our study, this warming intensity is only relevant for the medium-term warmed grassland soils (+ 9°C), but not for the long-term warmed grassland soils, the medium-term warmed forest soils, and the short-term warmed grassland soils. Furthermore, our earlier published metatranscriptomics studies [6, 17] indicated few differences in community composition between non-warmed and warmed soils (July 2016 samples), and the observed downregulation of the bacterial protein biosynthesis machinery in response to warming was taxonomically widespread, including community members that did not change in relative abundance in response to warming [17]. Additionally, reanalysing these metatranscriptomics datasets did neither reveal significant differences in rRNA operon copy number corrected relative abundances of bacterial taxa nor estimated community mean rRNA operon copy numbers at the grassland sites (Supplementary Tables S17 and S18), suggesting that the lower RNA:DNA and RNA:MBC ratios in the warmed soils were not related to compositional community changes towards bacteria with low rRNA operon copy numbers. Thus, ribosome reduction likely represents a physiological adjustment of the microbial community and not the physiological state of an altered and adapted community.

**Figure 3:**
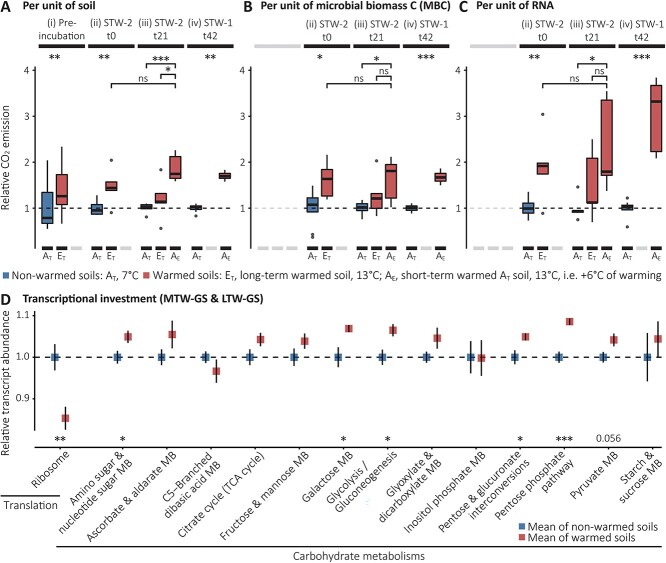
CO_2_ emissions from grassland soil incubations and *in situ* transcriptional investments. (A) Boxplots showing relative differences in soil CO_2_ emission rates of long-term (E_T_) and short-term (A_E_) warmed grassland soils (both in red) and their non-warmed control counterparts (A_T_; blue) calculated from measurements of CO_2_ accumulations over 24 h (mean of A_T_ = 1). (**i**) During the 3-week preincubation period of STW-2 (24 h accumulations 19, 12, and 5 days before the start of the experiment are summarized). (**ii**) After the 3-week preincubation period of STW-2; before short-term warming was started (at t0). (**iii**) At the end of STW-2, after 3 weeks of warming (t21). (**iv**) At the end of STW-1, after 6 weeks of warming (t42; LTW-GS and MTW-GS summarized). See Supplementary Information and Supplementary Fig. S2 for details on the experimental setups. (B) Same as (A) but showing relative differences in CO_2_ emissions per unit of microbial biomass C. (C) Same as (A) but showing relative differences in CO_2_ emissions per unit of RNA. Two-sided *t* tests were performed to test for significant differences; ^*^P < .05, ^*^^*^P < .01, ^*^^*^^*^P < .001; see Supplementary Table S19 for details and exact *P* values. (D) Relative abundances of transcripts assigned to KEGG carbohydrate metabolisms and the “translation” subcategory “ribosome” at the MTW-GS and LTW-GSs (both sites summarized; July 2016 samples; mean of A_T_ = 1). Two-sided *t* tests were performed to test for significant differences between warmed soils (red) and their non-warmed counterparts (blue); ^*^P < .05, ^*^^*^P < .01, ^*^^*^^*^P < .001; see Supplementary Table S20 for details and exact *P* values. Error bars represent standard error of the mean.

Even if some causes and mechanisms behind ribosome reduction and the exact contribution of different drivers remain to be elucidated, our observations demonstrate that subarctic soil microorganisms are depleted of ribosomes under short-, medium-, and long-term warming. Ribosome reduction appears to be a consistent response to warming and might offer a selective advantage at higher temperatures. Furthermore, especially in the context of global warming and changes in seasons such as longer summers and shorter winters [34], ribosome reduction may have far-reaching ecological consequences. For example, microbial resource reallocation enabled by ribosome reduction could affect soil C cycling. To explore this possibility, we assessed the link between CO_2_ emissions and the soil content of MBC and RNA in incubation experiments (Fig. 3A–C, Supplementary Fig. S2). Monitoring of CO_2_ emissions per unit of soil during the 3-week preincubation periods prior to the short-term warming experiments revealed significantly higher CO_2_ emissions in the long-term warmed soils (E_T_) compared to their non-warmed counterparts (A_T_) (Fig. 3A-i); both incubated at their mean *in situ* temperature of the sampling month (October). At the end of the 3-week preincubation period, CO_2_ emissions per unit of soil were 1.5 times higher in E_T_ than A_T_ (Fig. 3A**-**ii), CO_2_ emissions per unit of MBC (Fig. 3B**-**ii) and per unit or RNA (Fig. 3C**-**ii) were 1.6 times, and 1.9 times higher in E_T_ than A_T_, respectively. Likewise, CO_2_ emissions of 3-week-warmed soils (A_E_) were significantly higher than the emissions of their non-warmed counterparts (A_T_) (Fig. 3A–C-iii), with 1.9, 1.6, and 2.4 times higher CO_2_ emissions per unit of soil, MBC, and RNA, respectively. After three more weeks, at the end of the 6-week warming experiments, CO_2_ emissions of the 6-week-warmed soils (A_E_) continued to be significantly higher than the emissions of their non-warmed counterparts (A_T_), with on average 1.7, 1.7, and 2.8 times higher CO_2_ emissions per unit of soil, MBC, and RNA, respectively (Fig. 3A–C-iv). However, clear indications for ribosome reduction were only observed after 6 weeks of warming (Fig. 1D, STW-1) but not after 3 weeks of warming (Supplementary Fig. S4, STW-2). Thus, although ribosome reduction is not necessary for increased CO_2_ emissions from warmed soils at the onset of warming, it is clearly not mitigating CO_2_ emissions either. Correspondingly, we observed higher microbial transcriptional investment in carbohydrate metabolisms in medium- and long-term warmed soils sampled in July 2016 compared to their non-warmed counterparts, contrasting lower transcriptional investment in ribosomes (Fig. 3D). These differences in transcriptional investments may be indicative of microbial resource reallocation associated with accelerated C cycling within microbial cells that could potentially affect soil C cycling. In accordance with this interpretation, an earlier study conducted on the soil temperature gradients of the LTW-GS reported significantly higher biomass-specific organic C uptake and respiration rates in soils warmed by +1.5°C and + 6°C compared to their non-warmed counterparts [12]. Further studies are needed to finally establish and quantify the links between ribosome reduction, resource reallocation, and microbial C cycling and eventually assess the consequences of microbial ribosome content adjustments for the terrestrial C cycle, but some indications of links between microbial resource allocation and carbon turnover have been observed. Recently it was shown that methanotrophs exposed to temperature change alter their resource allocations, protein biosynthesis machinery adjustments being central in this physiological shift, with impact on the amount of methane consumed per cell division [35]. It has also been suggested that physiological responses to environmental changes and stresses can result in altered ecosystem-level C, energy, and nutrient flows [36]; e.g. the authors calculated that, at a conservative estimate, 3–6% of the annual net primary production in a grassland ecosystem can be consumed by microorganisms during a single drought episode to build up the osmolytes needed to survive. It is therefore conceivable that fluctuations in microbial ribosome contents, in the range indicated here (e.g. ribosome contents twice as high in winter compared to summer, Fig. 1E), may have comparable impacts.

### Further potential implications of ribosome reduction

Ribosomes account for up to ~40% of total bacterial cell dry mass (based on [37–39]). Thus, ribosome reduction also means that each cell carries less biomass, with direct influence on the amount of matter and energy required to build and maintain a cell and possibly also the total amount of microbial biomass present in a system, if the population size (i.e. cell numbers) stay constant. This also suggests the possibility of a temperature-driven reduction in cell sizes, as for example observed in pure cultures [35, 40] and marine prokaryotic communities [41] because smaller cells might carry the fewer ribosomes. Decreasing cell sizes further relate to higher surface to volume ratios that would positively affect nutrient uptake and distribution within cells [42]. Thus, ribosome reduction in combination with cell size reduction may offer the advantage of lower cellular operating costs in long-term warmed soils that are often characterized by lower substrate availabilities [7, 8]. Furthermore, ribosomes can be seen as storage compounds with high nutritional value that become scarce in warmed soils. Thus, ribosome reduction may also affect soil microbial food webs and trophic interactions, by affecting the substrate availability and quality for both bacterivorous microorganisms and necromass degraders, which in turn could drive unexpected soil C cycling responses [1]. Moreover, temperature-driven adjustments of cellular ribosome contents could be part of the explanation why microbial biomass in temperate, boreal, and arctic soil ecosystems often peaks in winter and decreases in spring again, a dynamic possibly linked to C stabilization in soil that is threatened by soil warming ([43] and references therein).

## Conclusion

In addition to ribosome content adjustments in soil microorganisms exposed to warming, temperature-driven adjustments of cellular ribosome contents have also been indicated in the phytoplankton model organism *Chlamydomonas reinhardtii* [44] and a range of poikilothermic organisms including plants and animals [45]. Thus, terrestrial and aquatic micro- and macro-biological responses to warming might commonly involve cellular ribosome adjustments. We, therefore, propose that ribosome content adjustments represent a mechanism for ecosystem-wide temperature acclimation that could substantially influence the effect of global warming on biogeochemical cycling.

## Supplementary Material

Supplementary_Information_final_wrae081

Supplementary_Tables_Revision_APRIL_wrae081

## Data Availability

All data needed to evaluate the results presented and the conclusions made in this study are included in this published article and its supplementary information files. Furthermore, all underlying data and scripts needed to replicate the presented analyses are available on DataverseNO (10.18710/OW27B6). Additionally, Supplementary Table S21 represents a collection of all data presented in this study. The raw sequence data are available at the NCBI Sequence Read Archive (SRA); BioProject ID: PRJNA663238, accession numbers SAMN16124403 – SAMN16124422.
