# Supplementary material for: Microorganisms in subarctic soils are depleted of ribosomes under short-, medium-, and long-term warming
Source: ISME J. 2024 May 9;18(1):wrae081. doi: 10.1093/ismejo/wrae081 (PMC11126301; doi:10.1093/ismejo/wrae081)
Supplement: Supplementary_Information_final_wrae081 [file supplementary_information_final_wrae081.pdf]

Supplementary Information and Supplementary Figures

Microorganisms in subarctic soils are depleted of ribosomes under short-, medium-, and long-term warming

Andrea Söllinger[1*], Laureen S. Ahlers[1], Mathilde Borg Dahl[2], Páll Sigurðsson[3‡], Coline Le Noir de Carlan[4], Biplabi Bhattarai[5], Christoph Gall[6], Victoria S. Martin[6], Cornelia Rottensteiner[6], Liabo L. Motleleng[1], Eva Marie Breines[1], Erik Verbruggen[4], Ivika Ostonen[5], Bjarni D. Sigurdsson[3], Andreas Richter[6], Alexander T. Tveit[1*]

[1] Department of Arctic and Marine Biology, UiT The Arctic University of Norway, Tromsø, Norway.

[2] Institute of Microbiology, University of Greifswald, Greifswald, Germany.

[3] Agricultural University of Iceland, Hvanneyri, Iceland.

[4] Research Group Plants and Ecosystems (PLECO), University of Antwerp, Wilrijk, Belgium.

[5] Department of Geography, University of Tartu, Estonia.

[6] Centre for Microbiology and Environmental Systems Science, University of Vienna, Vienna, Austria.

[‡] Current affiliation: Icelandic Forest Service, Selfoss, Iceland.

*Corresponding authors:

Andrea Söllinger (andrea.soellinger@uit.no)

Alexander T. Tveit (alexander.t.tveit@uit.no)

Supplementary Information

(i.e. extended "Materials and Methods")

ForHot grassland sites

The ForHot *Experiment* [1] in Iceland, located near Hveragerði (64°0'N, 21°11'W), represents the longest lasting *in situ* warming experiment worldwide and comprises replicated soil temperature gradients resulting from geothermal activities (**Supplementary Figure S1**). Five stable natural soil temperature gradients were established in an area covered by natural grassland that has been partly warmed for more than 50 years (long-term warmed grassland site = LTW-GS). The area has likely been warmed since before the 18th century, with varying geothermal activities over time, but stable conditions are prevalent since at least 1963 (ref. [1]). Nearby, in the same natural grassland area, five new temperature gradients emerged after an earthquake in 2008 (ref. [1]) (medium-term warmed grassland site = MTW-GS). Besides the MTW-GS, a Sitka spruce forest, planted in 1966, was affected by the earthquake in 2008, and five additional soil temperature gradients were established [1] (medium-term warmed forest site = MTW-FS). All gradients include non-warmed control plots (A_T) and warmed plots with an anticipated temperature increase of +6°C (E_T); between A_T and E_T plots are three more plots B_T, C_T, and D_T, with intermediate warming intensities (**Supplementary Figure S1**). On all plots, soil temperatures at 10 cm depth have been hourly logged since 2013, showing stable levels of soil warming throughout seasons (**Figure 1B**), but also revealing a significantly higher mean hourly temperature difference between A_T and E_T plots at MTW-GS (+8.8°C) compared to LTW-GS (+5.9°C) (two-sided t.test, n = 639 974, t = 283.41, $p < 2.2 \times 10^{-16}$). The E_T plots at MTW-FS are partly damaged due to dying and falling trees from adjacent plots with elevated warming (> +6°C of warming). Selecting E_T and A_T plots was pragmatical, as a much broader range of context data was available for the grassland E_T and A_T plots than other plots. To avoid dealing with the interference of dying trees on the temperature effect, the MTW-FS-E_T plots were replaced by D_T plots, which showed a mean hourly temperature difference to the non-warmed control plots of +2.8°C (**Figure 1B**). The soils of all three sites are classified as Silandic Andosols and the vegetational cover of the grasslands is dominated by

Agrostis capillaris, whereas the forest is dominated by *Picea sitchensis*, with no significant understorey vegetation [1].

Soil sampling and processing

Soil sampling campaigns for the seasonal survey (i.e. *in situ* study) and two short-term warming experiments were conducted at seven different timepoints throughout several years representing different seasons (**Figure 1A**, **Supplementary Table S1**), i.e., in July 2016 (ref. [2, 3]) (summer: LTW-GS and MTW-GS), October 2020 (autumn: LTW-GS and MTW-GS; and first short-term warming experiment (STW-1)), April 2021 (spring: LTW-GS and MTW-GS), October 2021 (autumn: MTW-FS; and second short-term warming experiment (STW-2)), February 2022 (winter: LTW-GS, MTW-GS, and MTW-FS), May 2022 (spring: MTW-FS), and July 2022 (summer: MTW-FS). ***Seasonal survey***: Soil samples (upper 10 cm) were taken from LTW-GS, MTW-GS, and MTW-FS A_T and E_T plots (GS) and A_T and D_T plots (FS) using a metal corer (~3 cm diameter); vegetational cover was removed. The summer 2016 samples were taken and processed as described previously [3] (flash frozen in liquid nitrogen), all other samples were flash frozen and transported on dry ice, ground in liquid nitrogen, and stored at -80°C until processing. ***First short-term warming experiment*** (STW-1): Approximately 300 g of soil (upper 10 cm) were sampled from non-warmed plots (A_T plots) of two ForHot gradients, i.e., MTW-1-A_T and LTW-4-A_T, in October 2020. Soil samples were transported on cool packs, sieved to 2 mm, and incubated at the approximated mean *in situ* October soil temperature of 7°C until experiment start. Additionally, soils from the corresponding long-term warmed plots (E_T plots) were sampled, processed the same way, and incubated at 13°C. ***Second short-term warming experiment*** (STW-2): Approximately 300 g of soil (upper 10 cm) were sampled from the non-warmed plots (A_T plots) of all five replicated long-term warmed grassland soil temperature gradients (LTW-GS), in October 2021; processed as described above, and incubated at the approximated mean *in situ* October soil temperature of 7°C until experiment start. Additionally, soils from the corresponding long-term warmed plots (E_T plots) were sampled, processed the same way, and incubated at 13°C to allow a comparison of short-, and long-term soil warming effects on microbial communities. See

Supplementary Table S1 for a detailed sample overview, exact sampling timepoints, sampling conditions, and *in situ* soil temperatures during sampling.

Short-term warming experiments

Two complementary short-term warming experiments were conducted (STW-1 and STW-2) using sieved grassland soils (2 mm mesh size) sampled from A_T and E_T plots in October 2020 and October 2021 (**Supplementary Table S1**). All microcosms were incubated in the dark throughout the pre-incubations and the main experiments. ***First short-term warming experiment*** (STW-1): Sieved grassland soils from two gradients, MTW-1-A_T and LTW-4-A_T, were separated into ten 100 mL serum bottles per gradient, each containing approximately 22 g of soil (**Supplementary Figure S2A**). After a pre-incubation of three weeks at the approximated mean *in situ* October soil temperature of 7°C, the ten replicates of each soil were split. Five replicates were further incubated at 7°C (control incubations), whereas the other five replicates were incubated at 13°C (+6°C of warming). All soil microcosm bottles were incubated for six weeks. Sub-samples (~1.5 g) for molecular analyses were taken at the beginning of the experiment (t0, right before bottling, "timepoint 0"), after three weeks of incubation (t21) and at the end of the incubation (t42), flash frozen, ground in liquid nitrogen, and stored at -80°C until further processing (**Supplementary Figure S2A**). Gravimetric water content (GWC) was determined at t0 and t42 by drying 2 g of soil (24 h at 100°C); DW, dry weight. Total carbon (C) and nitrogen (N), total dissolved organic C (DOC) and N (TDN), and microbial biomass C (MBC) and microbial biomass N (MBN) contents were analysed as described previously[3] and below. Sieved soil from MTW-1-E_T and LTW-4-E_T, respectively, separated into five 100 mL serum bottles, each containing approximately 22 g of soil, acted as long-term warmed controls (**Supplementary Figure S2A**). Sub-samples for molecular analysis, GWC, and total and microbial biomass C and N were taken after three weeks of incubation (i.e., the pre-incubation time described above) at the approximated mean *in situ* long-term warming October soil temperature of 13°C and processed as described above. ***Second short-term warming experiment*** (STW-2): Sieved soils from A_T and E_T plots of all five replicated long-term warmed grassland soil temperature gradients (LTW-GS) were bottled (**Supplementary Figure S2B**). The

five A_T soils were each separated into two 500 mL glass bottles, containing approximately 100 g of soil, whereas only one bottle per E_T soil was prepared, containing also approximately 100 g of soil. The resulting 15 bottles were pre-incubated for three weeks at their respective approximated mean *in situ* October soil temperature, i.e., 7°C (A_T soil bottles) and 13°C (E_T soil bottles). After the pre-incubation, one set of A_T microcosms was exposed to +6°C of warming, resulting in an incubation temperature of 13°C ("Short-term warming at E_T"), whereas the other set of A_T microcosms was kept at 7°C ("Non-warmed control at A_T"). The E_T microcosms were kept at 13°C ("Long-term warming control at E_T"). All soil microcosm bottles were incubated for three weeks. Sub-samples (~1.5 g) for molecular analysis were taken at the beginning of the experiment (t0) and after three weeks of incubation (t21), flash frozen, ground in liquid nitrogen, and stored at -80°C until further processing (**Supplementary Figure S2B**). Gravimetric water content was determined at t0 and t21 by drying 2 g of soil (24 h at 100°C); DW, dry weight. Soil pH at t0 and t21 was measured at room temperature using 2 g of fresh soil suspended in 5 mL of a 0.05 M $CaCl_2$ solution. Total C and N, total dissolved organic C (DOC) and N (TDN), and microbial biomass C (MBC) and microbial biomass N (MBN) contents were analysed as described previously [3] and below.

Gas chromatography

Soil CO_2 emission rates (nM CO_2 h^{-1} g^{-1} DW soil) of both short-term warming experiments (**Supplementary Figure S2**) were obtained by measuring 24 h CO_2 accumulations in the microcosm serum bottles regularly throughout the experiments using a gas chromatograph (SRI 8610C, SRI Instruments; equipped with a flame ionising detector) and inferring CO_2 concentrations via standard curves created from gases with known CO_2 concentrations applying the general gas equation. During the 24 h periods serum bottles were sealed with air-tight rubber stoppers; between measurements the bottles were aerated and closed with aluminium foil.

Total nucleic acid extractions

DNA and RNA contents were obtained by extracting total nucleic acids (TNA) from flash frozen soil samples taken on site (seasonal survey) or during the incubation experiments (STW-1 and STW-2) using a quantitative phenol-chloroform extraction protocol to allow a comparison of non-warmed (A_T) and warmed soil (E_T, D_T) soils within and between seasons and incubation experiments. Prior to TNA extractions the flash frozen samples were ground in liquid N and homogenised. See [3] and **Supplementary Table S2** for details on the quantitative extraction protocol. Total RNA and DNA contents were quantified using a Qubit 2.0 Fluorometer (Thermo Fisher Scientific, Waltham, MA, USA) and the Qubit RNA HS Assay Kit and the Qubit dsDNA HS Assay Kit, respectively.

Biological and physicochemical soil properties

Seasonal survey **(i.e.** ***in situ*** **study*)***: Gravimetric water content was determined immediately after soil sampling by drying 2 g of soil (24 h at 100°C); DW, dry weight. C and N compounds were extracted and quantified using standard procedures employed previously [3]. Briefly, total C and N contents were analysed in dried soil aliquots using an elemental analyser coupled to an isotope ratio mass spectrometer (EA-IRMS; EA1110 coupled via a ConFlo III interface to a DeltaPLUS IRMS, Thermo Fisher Scientific). Total dissolved organic C (DOC) and N (TDN) concentrations were obtained in KCl extracts (in 1:5 dilutions with water) on a DOC/TDN analyser (Shimadzu TOC-VCPH/CPNTNM-1 analyser, Kyoto, Japan), after extracting 2 g fresh soil aliquots with 15 mL of a 1 M KCl solution for 30 min at room temperature. Microbial biomass C (MBC) and microbial biomass N (MBN) contents were determined via a chloroform-fumigation extraction method after [4] (48 h incubation period of 2 g fresh soil aliquots followed by KCl extraction, as described above), and calculated as the difference between fumigated samples and non-fumigated controls. Thus, the presented MBC and MBN contents represent the extractable fraction of the total microbial biomass C and N. Fine root biomass (mg dry roots g^{-1} DW soil) was obtained from a second set of soil cores sampled in parallel and next to the soil cores used for total nucleic acid extractions in October 2020 and April 2021. After being freeze-dried, soil-free fine roots (< 0.5 mm) were collected in a timed and standardised way across samples.

Short-term w*arming experiments*: Biological and physicochemical soil properties were measured using the same methods and protocols as described above; see section "Short-term warming experiments" and **Supplementary Figure S2** for details on the sampling timepoints.

***Extrapolation of microbial biomass C content in grassland soils*:** We evaluated the possibility of extrapolating microbial biomass C (MBC) contents from microbial biomass N (MBN) contents because C contaminated filters used in the second short-term warming experiment (STW-2) rendered the calculation of MBC from DOC measurements impossible. First, we analysed differences in MBC:MBN ratios between temperature groups (A_T vs. E_T), grassland sites (MTW-GS vs. LTW-GS), warming durations (A_T control vs. 21 days vs. 42 days vs. 8 years vs. > 50 years), sampling month (July vs. October vs. February), and sampling years (2016 vs. 2020 vs. 2022) using all grassland samples (n=74) with measured DOC and TDN and resultant MBC and MBN contents (**Supplementary Table S3**). No significant differences in MBC:MBN ratios were observed (**Supplementary Table S3**). Thus, we next tested the relationship between MBC and MBN contents and revealed a strong positive linear correlation (Pearson's product-moment correlation, n = 65, r = 0.94, p value < 2.2×10^{-16}; **Supplementary Figure S3A**). We excluded the July 2022 samples (n = 9) as no other extractions or analyses were performed on those samples and we therefore used them to test linear (mixed) models generated from the remaining dataset (n = 65). Excluding the July 2022 samples had no significant effect on the Pearson's product-moment correlation (including July 2022: n = 74, r = 0.92, p value < 2.2×10^{-16}). Linear (mixed) models were generated taking random effects of temperature group, grassland site, warming duration, sampling month, and sampling year into account. R-square values ranged from 0.89 – 0.98 (**Supplementary Table S4**). Model performances were evaluated using Akaike Information Criterion (**Supplementary Table S4**) and tested on the July 2022 samples (**Supplementary Table S4b, Supplementary Figure S3B**). Comparing the ratios of RNA to MBC and MBN, respectively, from the first short-term warming experiment on LTW grassland soils, revealed a highly similar pattern that underpinned the applicability, indicated above, of extrapolating MBC contents from MBN contents

measured in the second short-term warming experiment on LTW grassland soils (**Supplementary Figure S3C**).

We employed Model #2 ($R^2 = 0.95$), which was slightly underestimating MBC contents at MBN contents < 50 µg N g^{-1} DW soil in our test dataset (**Supplementary Figure S3B**, **Supplementary Table S4**) to extrapolate MBC contents in the second short-term warming experiment (**Supplementary Table S5**). As 93% of the MBN contents in the second short-term warming experiment were < 50 µg N g^{-1} DW soil, this model provided a conservative estimate. The extrapolated MBC contents were used to calculate RNA:MBC ratios and reveal if RNA contents per unit of MBC are lower in warmed soils.

Data analyses

Seasonal survey* (i.e. *in situ* study)**: To compare differences in RNA:DNA ratios between non-warmed control plots (A_T) and medium- and long-term warmed plots within and across sites and seasons we calculated standardised values setting the mean of A_T replicates per site and timepoint to 1 and calculating the relative differences to the mean of these A_T replicates (i.e., showing the fold-change). Thus, warmed soils with values > 1 show an increase in RNA:DNA ratios compared to their non-warmed counterparts, whereas warmed soils with values < 1 show a decrease in RNA:DNA ratios compared to their non-warmed counterparts. ***Short-term warming experiments: Mean relative RNA:DNA ratios were calculated as described above. Relative CO_2 emissions were calculated likewise. ***Correlation matrix***: Correlations (Spearman's rank correlations) between biological and physicochemical parameters were calculated using all available timepoints from the seasonal survey and the short-term warming experiments that included RNA and MBC values. Depending on the parameter the number of observations, n, ranged from 26 – 115 (see **Supplementary Table S9** for details).

Metatranscriptomics: See main text.

R packages & figure design: We used Rstudio (rstudio.com) and R (r-project.org), version 4.2.2, to analyse the data, perform statistical tests, and graphically display the results including the R packages tidyverse (includes ggplot2 version 3.3.6) (tidyverse.tidyverse.org) version 1.3.1, gridExtra (cran.r-

project.org/web/packages/gridExtra) version 2.3, reshape2 (github.com/hadley/reshape) version 1.4.4, plyr (had.co.nz/plyr) version 1.8.7, maps (https://cran.r-project.org/web/packages/maps) version 3.4.0, mapdata (https://cran.r-project.org/web/packages/mapdata) version 2.3.0, lme4 (github.com/lme4/lme4) version 1.1-31, performance (easystats.github.io/performance) version 0.10.0, Hmisc (hbiostat.org/R/Hmisc) version 4.7-1, corrplot (github.com/taiyun/corrplot) version 0.92, and multcompView (cran.r-project.org/web/packages/multcomp) version 0.1-8. Further details on the R packages and employed functions can be found in the respective sections and within the scripts available on DataverseNO (https://doi.org/10.18710/OW27B6). Adobe Illustrator was used for final figure editing.

Statistics: Hypothesising that RNA:DNA ratios are lower in warmed soils, one-sided t-tests (using the basic R function *t.test*) were performed to test for significant differences between non-warmed control soils and short-, medium- and long-term warmed soils at each timepoint of the seasonal survey and the short-term warming experiments (**Figure 1C – E**). Pairwise t-tests (using the basic R function *pairwise.t.test*) were used to test for significant differences in RNA:DNA ratios of the consecutively sampled non-warmed forest soils (**Figure 1F**). The Benjamini-Hochberg procedure (p.adjust.method = "BH") was used to correct *p* values for multiple testing. Correlations between biological and physicochemical soil parameters were investigated using the basic R function *cor.test* (method = "spearman"). Two-sided t-tests (*t.test*) were performed to test for significant differences in CO_2 emission rates from non-warmed, long-term, and short-term warmed soil incubations at different timepoints (**Figure 3A – C**), as well as significant differences in water contents, substrate availabilities, and fine root biomass between non-warmed and warmed soils (**Supplementary Figures S7 – S10**). Pairwise t-tests (using the basic R function *pairwise.t.test*) were used to test for significant differences in fine root biomass between seasons. The Benjamini-Hochberg procedure (p.adjust.method = "BH") was used to correct *p* values for multiple testing. Multiple linear regression models (using the basic R function *lm*) were used to test the effect of multiple distinct predictor variables on RNA:MBC and RNA:DNA ratios. Pairwise interactive effects of temperature and other environmental variables were

evaluated by integrating interactive effects in analysis of variance (ANOVA) models (using the basic R function *aov*). Two-sided t-tests (*t.test*) were employed to test for significant differences in copy-number corrected relative abundances of bacterial taxa and relative transcriptional investments of the soil microbial communities between non-warmed and warmed soils.

References

1. Sigurdsson BD, Leblans NIW, Dauwe S et al. Geothermal ecosystems as natural climate change experiments: The ForHot research site in Iceland as a case study. *Icelandic Agric Sci* 2016;29(1):53–71. https://doi.org/10.16886/IAS.2016.05.
2. Séneca J, Söllinger A, Herbold CW et al. Increased microbial expression of organic nitrogen cycling genes in long-term warmed grassland soils. *ISME Commun* 2021;1(1):69. https://doi.org/10.1038/s43705-021-00073-5.
3. Söllinger A, Séneca J, Dahl MB et al. Down-regulation of the bacterial protein biosynthesis machinery in response to weeks, years, and decades of soil warming. *Sci Adv* 2022;8(12):3230. https://doi.org/10.1126/sciadv.abm3230.
4. Vance ED, Brookes PC, Jenkinson DS. An extraction method for measuring soil microbial biomass C. *Soil Biol Biochem* 1987;19(6):703–7. https://doi.org/10.1016/0038-0717(87)90052-6.

Supplementary Figures

Supplementary Figure S1. ForHot sampling sites.

Supplementary Figure S2. Experimental setup of short-term warming experiments.

Supplementary Figure S3. Extrapolation of microbial biomass carbon (MBC) contents from microbial biomass nitrogen (MBN) contents in grassland soils.

Supplementary Figure S4. RNA to DNA ratios of the second short-term warming experiment (STW-2).

Supplementary Figure S5. Relationship between RNA contents and microbial biomass nitrogen (N) and DNA contents.

Supplementary Figure S6. Relationships between RNA contents per unit of microbial biomass carbon (MBC) and physicochemical soil parameters.

Supplementary Figure S7. *In situ* soil water contents and water content changes during incubation.

Supplementary Figure S8. Temperature, ribosome content proxies, soil water content, and carbon (C) and nitrogen (N) contents.

Supplementary Figure S9. Temperature, relative and total RNA contents, and carbon (C) and nitrogen (N) availabilities.

Supplementary Figure S10. RNA:DNA ratios and fine root biomass.

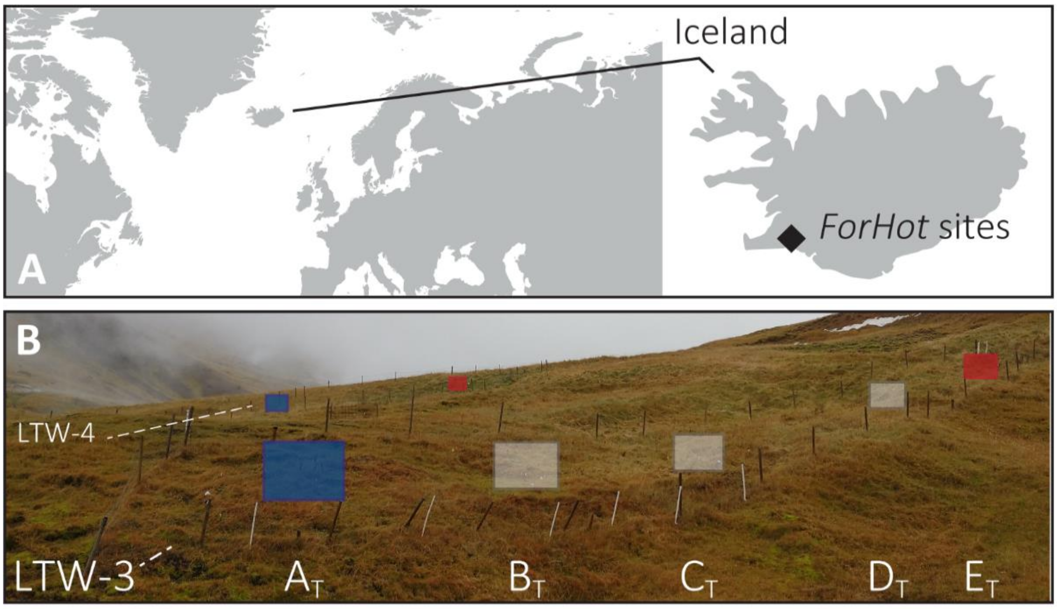

Supplementary Figure S1. ForHot sampling sites. (**A**) The ForHot project in Iceland represents the longest lasting *in situ* warming experiment worldwide and comprises replicated soil temperature gradients resulting from geothermal activities, some of which have been partly warmed for more than 50 years. (**B**) LTW-3 (i.e., LTW-GS-3) represents one out of 15 replicated natural soil temperature gradients (i.e., 5 gradients at the medium-term warmed grassland site (MTW-GS), 5 gradients at the long-term warmed grassland site (LTW-GS), and 5 gradients at the medium-term warmed forest site (MTW-FS). A_T plots represent non-warmed control plots, B_T and C_T plots are warmed by around +0.5°C to +2.0°C, D_T plots are warmed by approximately +3°C, and E_T plots are warmed by approximately +6°C (LTW-GS) and +9°C (MTW-GS). See **Supplementary Information** "ForHot grassland sites", Sigurdsson *et al*. 2016 (ref. [1]), and forhot.is for more information on the ForHot sites.

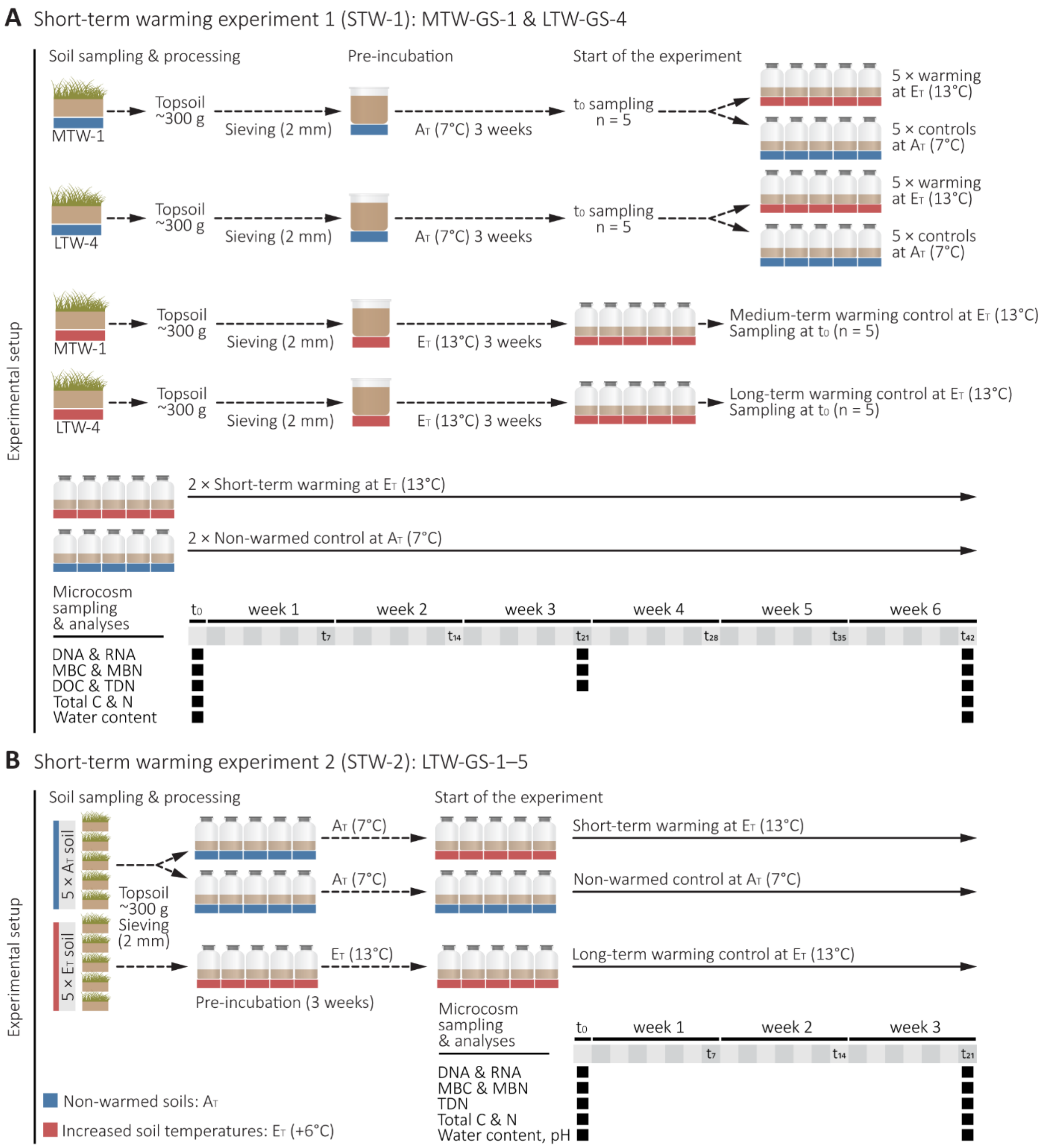

Supplementary Figure S2. Experimental setup of short-term warming experiments. (**A**) STW-1: Non-warmed soils from two ForHot temperature gradients were incubated at increased temperatures (+6°C) and sampled after 21 (t21) and 42 (t42) days of incubation. Short-term warmed soils were compared to non-warmed controls (parallel incubations) and medium and long-term warmed controls (sampled at t0). (**B**) STW-2: Non-warmed soils from all 5 replicated soil temperature gradients of the long-term warmed grassland site (LTW-GS) were incubated at increased temperatures (+6°C) and sampled after 21 days (t21) of incubation. The short-term warmed soils were compared to non-warmed controls (*Non-warmed control at* A_T) and long-term warm controls (*Long-term warming control at* E_T). Abbreviations: MTW-GS, medium-term warmed grassland site; LTW-GS, long-term warmed grassland site; C, carbon; N, nitrogen; MBC, microbial biomass C; MBN, microbial biomass N; DOC, dissolved organic C; TDN, total dissolved N. STW-1: 100 mL bottles a 22 g, STW-2: 500 bottles a 100 g soil.

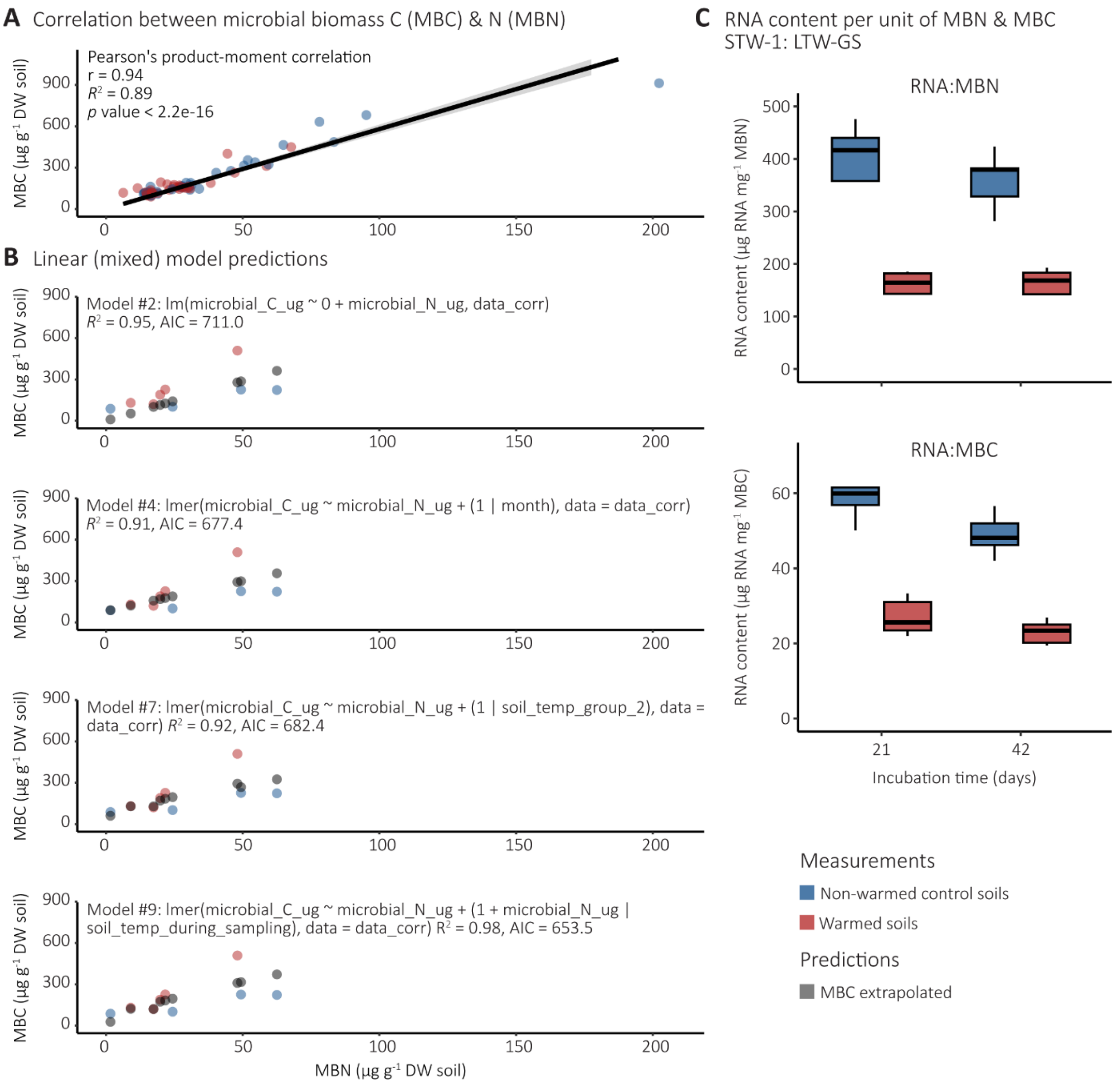

Supplementary Figure S3. Extrapolation of microbial biomass carbon (MBC) contents from microbial biomass nitrogen (MBN) contents in grassland soils. (**A**) MBC and MBN showed a strong positive linear correlation (Pearson's product-moment correlation, n = 65, r = 0.94, *p* value < 2.2×10^{-16}). (**B**) Measured MBC contents (blue and red dots) and predicted (grey dots) MBC contents using one linear model (Model #2) and three linear mixed models (Model #4, #7, and #9). Model #2, which was rather slightly under- than overestimating MBC contents at MBN contents < 50 µg N g^{-1} DW (dry weight) soil, was used to extrapolate MBC contents from MBN contents measured in the second short-term warming experiment (STW-2). (**C**) RNA contents per unit of MBN and MBC, respectively, obtained after 21 and 42 days of incubating LTW-GS samples at control (7°C) and elevated (13°C) soil temperatures (first short-term warming experiment; STW-1; **Supplementary Figure 2A**) revealed a highly similar pattern, and underpinned the applicability to extrapolate MBC contents from MBN contents measured in the second short-term warming experiment on LTW grassland soils. See **Supplementary Table S3** for all underlying data and **Supplementary Table S4 and S4b** for linear (mixed) models and their evaluation.

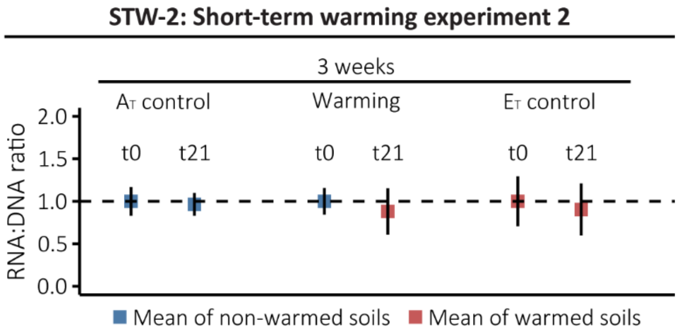

Supplementary Figure S4. RNA to DNA ratios of the second short-term warming experiment (STW-2). Mean relative RNA:DNA ratios of non-warmed controls (all 5 LTW-GS gradients; “A_T control”), warmed grassland soils (3 weeks warming of LTW-GS-A_T (all 5 gradients; “Warming”)), and long-term warmed soils (all 5 gradients; “E_T control”). Each treatment (“A_T control”, “Warming”, and “E_T control”) was normalised to the RNA:DNA ratio at the starting timepoint (t0), revealing that the experimental setup (**Supplementary Figure S2B**) itself and 3 weeks of warming (t21) is not significantly affecting RNA:DNA ratios in the second short term-term warming experiment. In contrast, significantly lower RNA:DNA ratios were observed after 3 weeks of warming in the first short-term warming experiment (STW-1) with LTW-GS-A_T soils, but not with MTW-GS-A_T soils (**Figure 1D**).

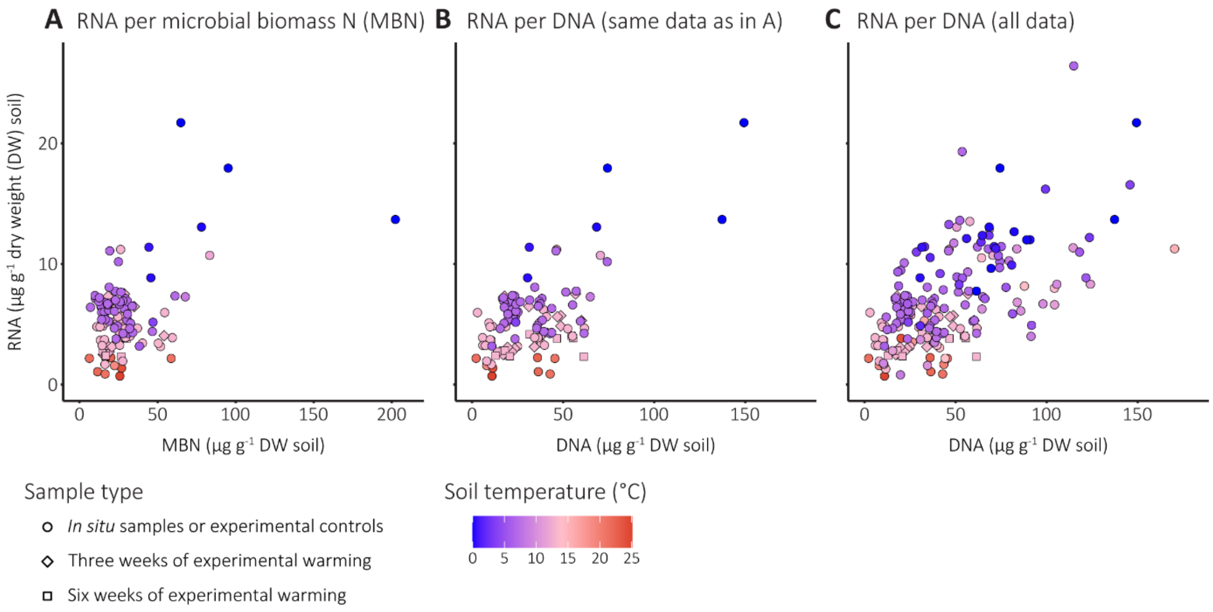

Supplementary Figure S5. Relationship between RNA contents and microbial biomass nitrogen (N) and DNA contents. (**A**) RNA content per unit of microbial biomass nitrogen (MBN) of all grassland soil samples with quantified MBN contents (*in situ* samples, short-term warming experiment 1, and short-term warming experiment 2; n = 115). RNA:MBN ratios and soil temperatures showed a moderate and significant negative correlation (Spearman's rank correlation, n = 115, r_s = -0.42, *p* value = 3.2×10^{-6}). (**B**) RNA content per unit of DNA of the same grassland soil samples as used in (**A**). RNA:DNA ratios and soil temperatures showed a moderate and significant negative correlation (Spearman's rank correlation, n = 115, r_s = -0.47, *p* value = 1.3×10^{-7}). (**C**) RNA content per unit of DNA of all grassland and forest samples. RNA:DNA ratios and soil temperatures of these extended dataset showed also a moderate and significant negative correlation (Spearman's rank correlation, n = 201, r_s = -0.37, *p* value = 7.3×10^{-8}). The correlation between RNA:DNA ratios and soil temperatures was slightly stronger if measurements from the short-term warming experiments were excluded and only *in situ* measurements from grassland and forest soils were considered (Spearman's rank correlation, n = 111, r_s = -0.40, *p* value = 1.1×10^{-5}).

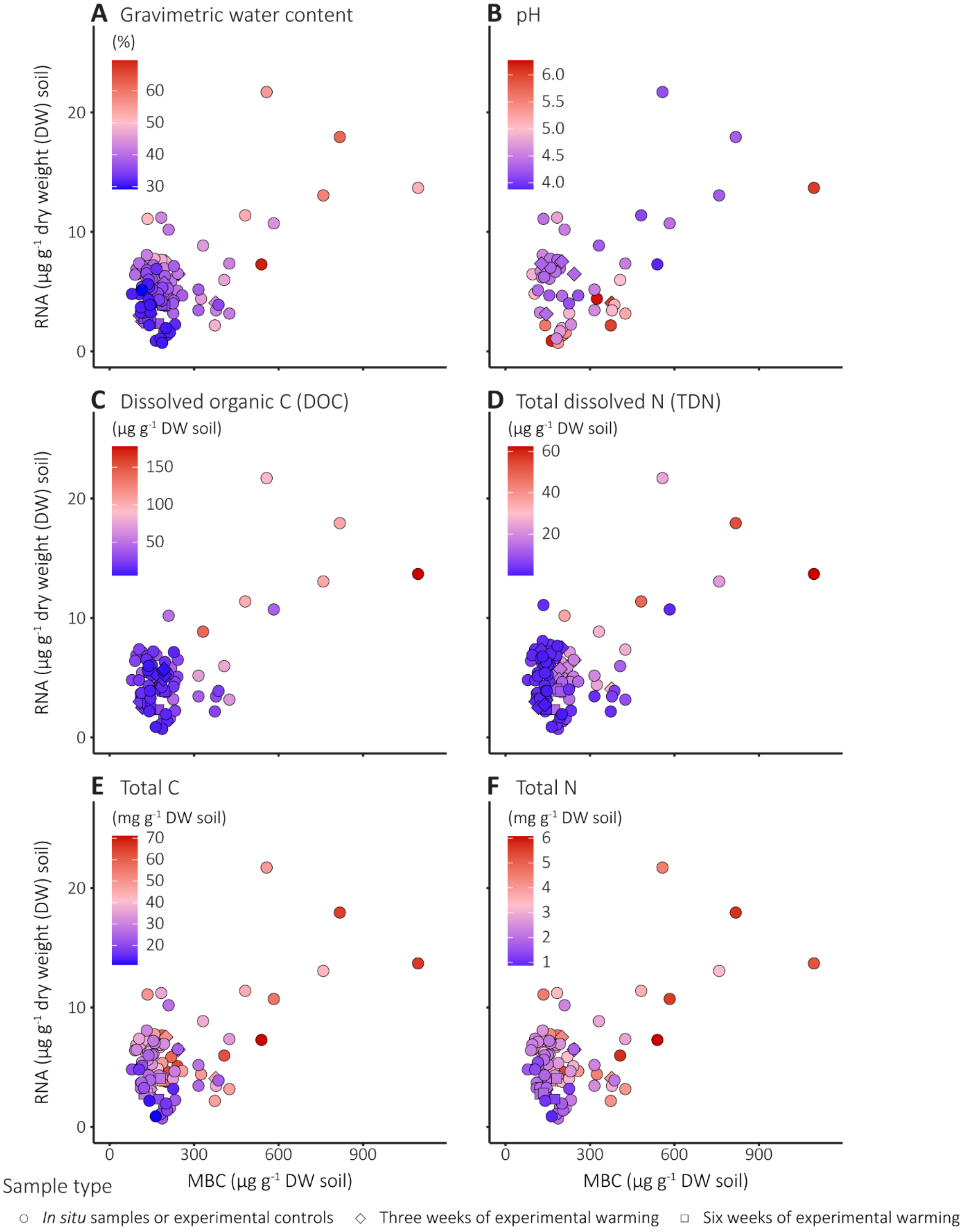

Supplementary Figure S6. Relationships between RNA contents, microbial biomass carbon (MBC) contents, and physicochemical soil parameters. Relationship between RNA contents, MBC contents, and soil water content (**A**), pH (**B**), dissolved organic C, DOC (**C**), total dissolved N, TDN (**D**), total C (**E**), and total N (**F**). One outlier with > 600 µg DOC and 130 µg TDN g^{-1} DW soil was removed from the mapping in (**C**) and (**D**) to enhance the colour code visualisation. Abbreviations: C, carbon; N, nitrogen; DW, dry weight; MBC, microbial biomass C; MBN, microbial biomass N.

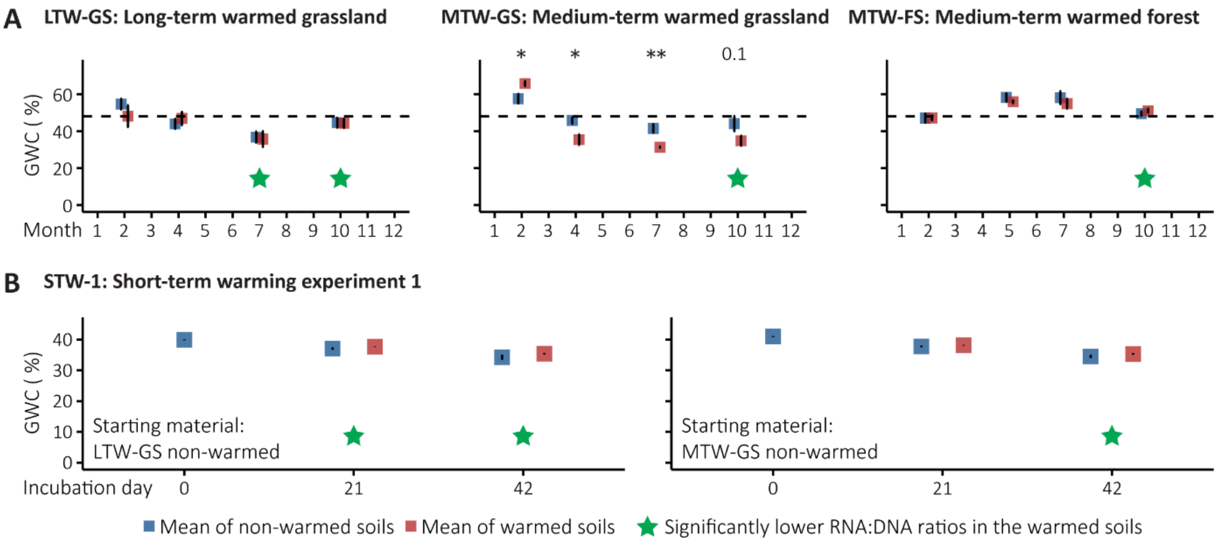

Supplementary Figure S7. *In situ* soil water contents and water content changes during incubation. (**A**) Mean gravimetric water contents (GWC) per site and sampling timepoint of non-warmed control soils (n = 5 per site; A_T; blue) and warmed soils (n = 5 per site; E_T and D_T; red). Two-sided t-tests were performed to test for significant differences; * = $p < 0.05$, ** = $p < 0.01$, *** = $p < 0.001$; MTW-GS October 2020: $p = 0.1$, indicating a trend towards lower soil water contents in the warmed soils at this timepoint; see **Supplementary Table S10** for details and exact p values. LTW-GS gradient 5 was only sampled in February 2022 (included here); if excluded from the analysis, soil water content is significantly lower in winter in the warmed LTW grassland soils compared to their non-warmed counterparts (see **Supplementary Figure S8**). Error bars represent standard error of the mean. The dashed line at 48% represents the mean soil water content of all sites and samples. (**B**) Mean gravimetric water contents (GWC) at the beginning of STW-1 (incubation day 0; n = 5) and after 21 and 42 days of incubation (n = 5 per timepoint, temperature, and site). Two-sided t-tests were performed to test for significant differences between warmed and non-warmed soils at the individual timepoints (no significant differences were detected; see **Supplementary Table S10** for details). Green stars highlight month and incubation days, respectively, with significantly lower RNA:DNA ratios in the warmed soils.

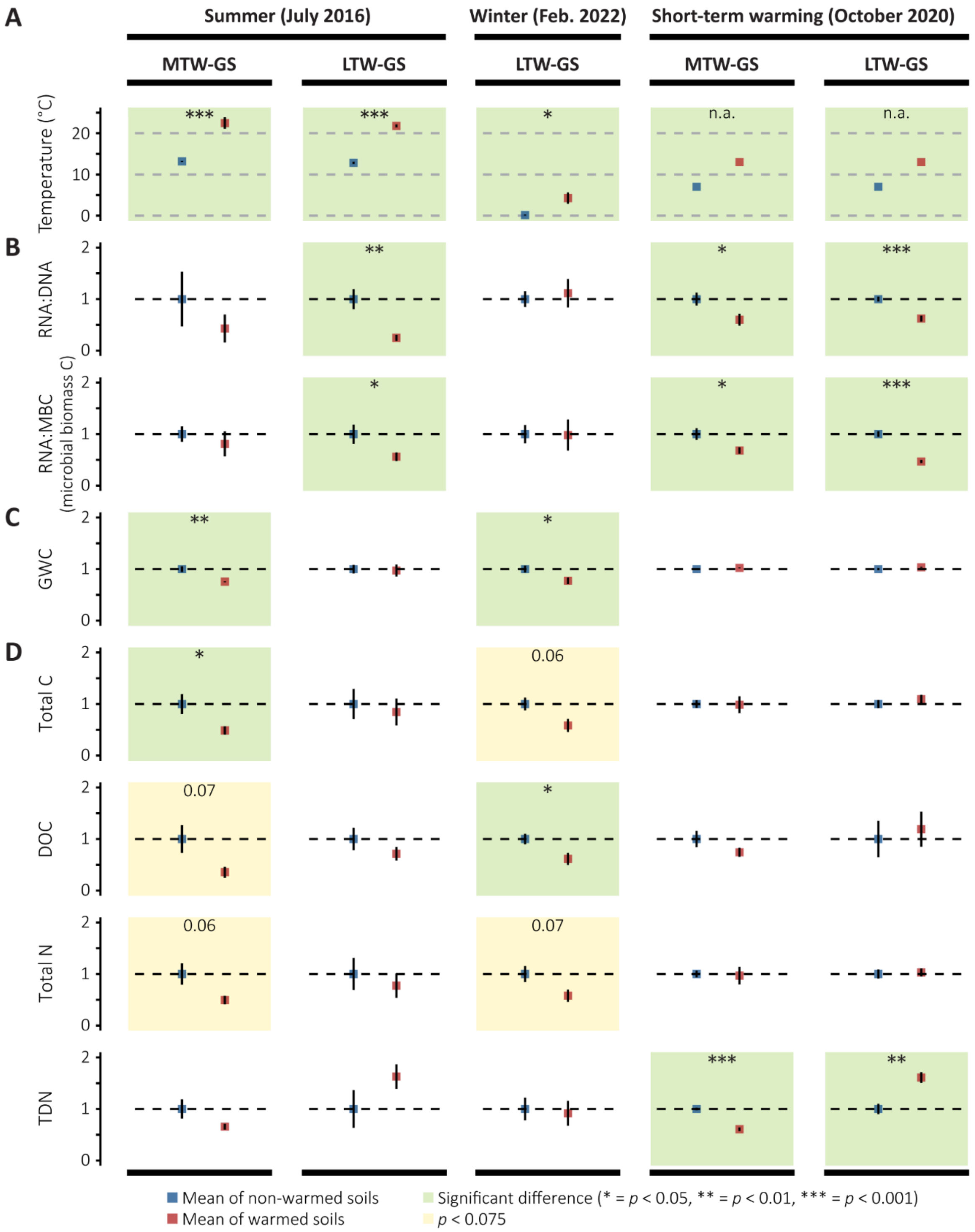

Supplementary Figure S8. Temperature, ribosome content proxies, soil water contents, and carbon (C) and nitrogen (N) contents. (**A**) Overview of the analysed *in situ* soil samples ("Summer" and "Winter" (excluding gradient 5)) and the endpoint samples of the first short-term warming experiment (STW-1: 6 weeks warming (+6°C) of non-warmed control soils (A_T), sampled in autumn 2020; see **Supplementary Figure S2A** for details) and mean soil temperatures during sampling (*in situ* samples) and during the short-term warming experiment. (**B**) Ribosome content proxies. (**C**) Gravimetric water contents (GWC). (**D**) Total C, dissolved organic C (DOC), total N, and total dissolved N (TDN) contents. (**B** – **D**) Ratios and contents are normalised by setting the mean of non-warmed control soils of each comparison to one (dashed line). Significant differences (t-test) are indicated with asterisks and colour code; see **Supplementary Table S11** for details and exact *p* values. Error bars = standard error of the mean. MTW-GS and LTW-GS, medium- and long-term warmed grassland sites.

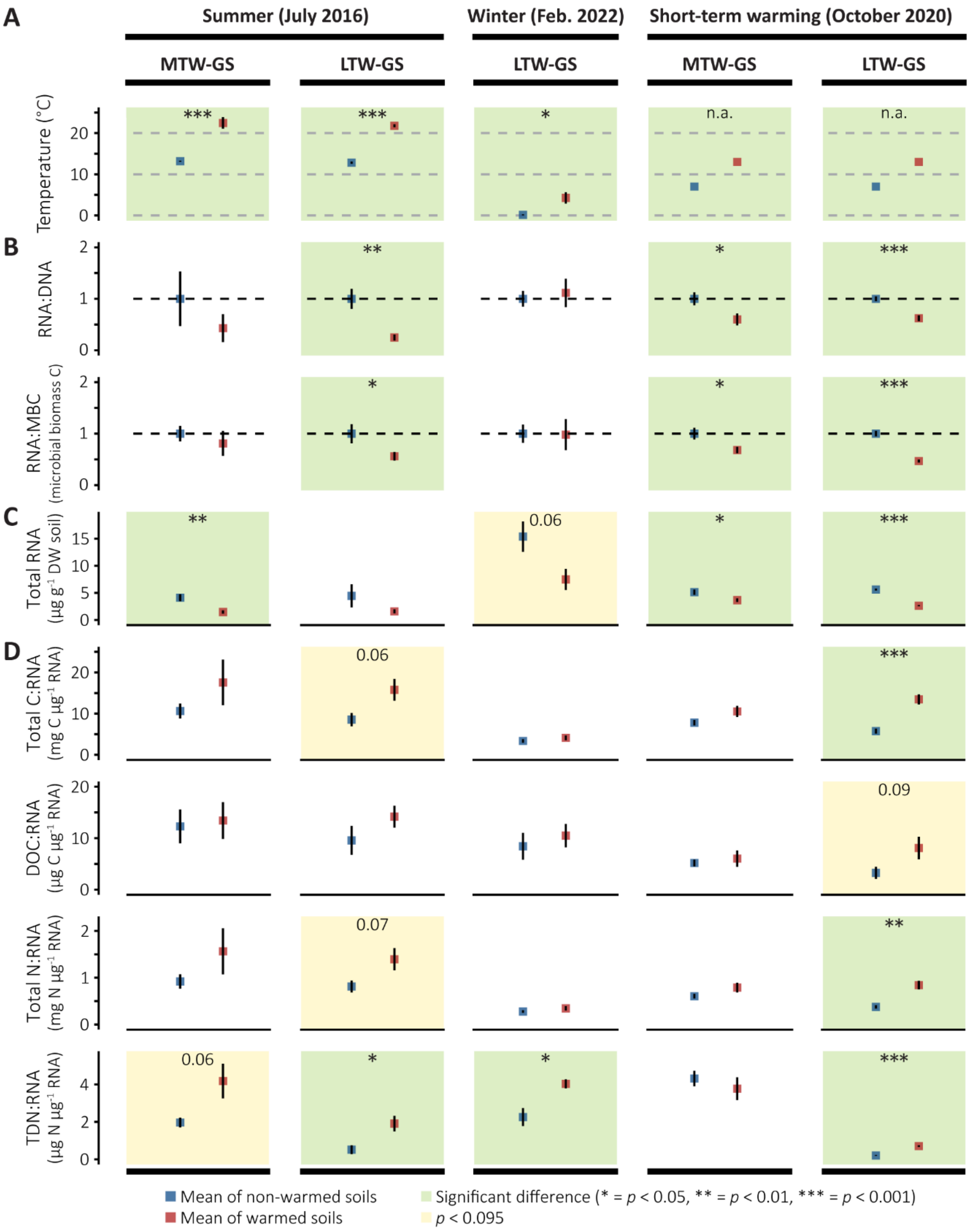

Supplementary Figure S9. Temperature, relative and total RNA contents, and carbon (C) and nitrogen (N) availabilities. (**AB**) same as **Supplementary Figure S8**: (**A**) Overview of the analysed *in situ* soil samples ("Summer" and "Winter" (excluding gradient 5)) and the endpoint samples of the first short-term warming experiment (STW-1: 6 weeks warming (+6°C) of non-warmed control soils (A_T), sampled in autumn 2020; see **Supplementary Figure S2A**) and mean soil temperatures during sampling (*in situ*) and during the short-term warming experiment. (**B**) Ribosome content proxies. (**C**) Total RNA content per g dry weight (DW) soil. (**D**) Ratios between total C, dissolved organic C (DOC), total N, and total dissolved N (TDN) contents and total RNA contents; higher values may indicate that substrate availabilities are not limiting ribosome synthesis. Significant differences (t-test) are indicated with asterisks and colour code; see **Supplementary Table S13** for details and exact *p* values. Error bars = standard error of the mean; MTW-GS and LTW-GS, medium- and long-term warmed grassland sites.

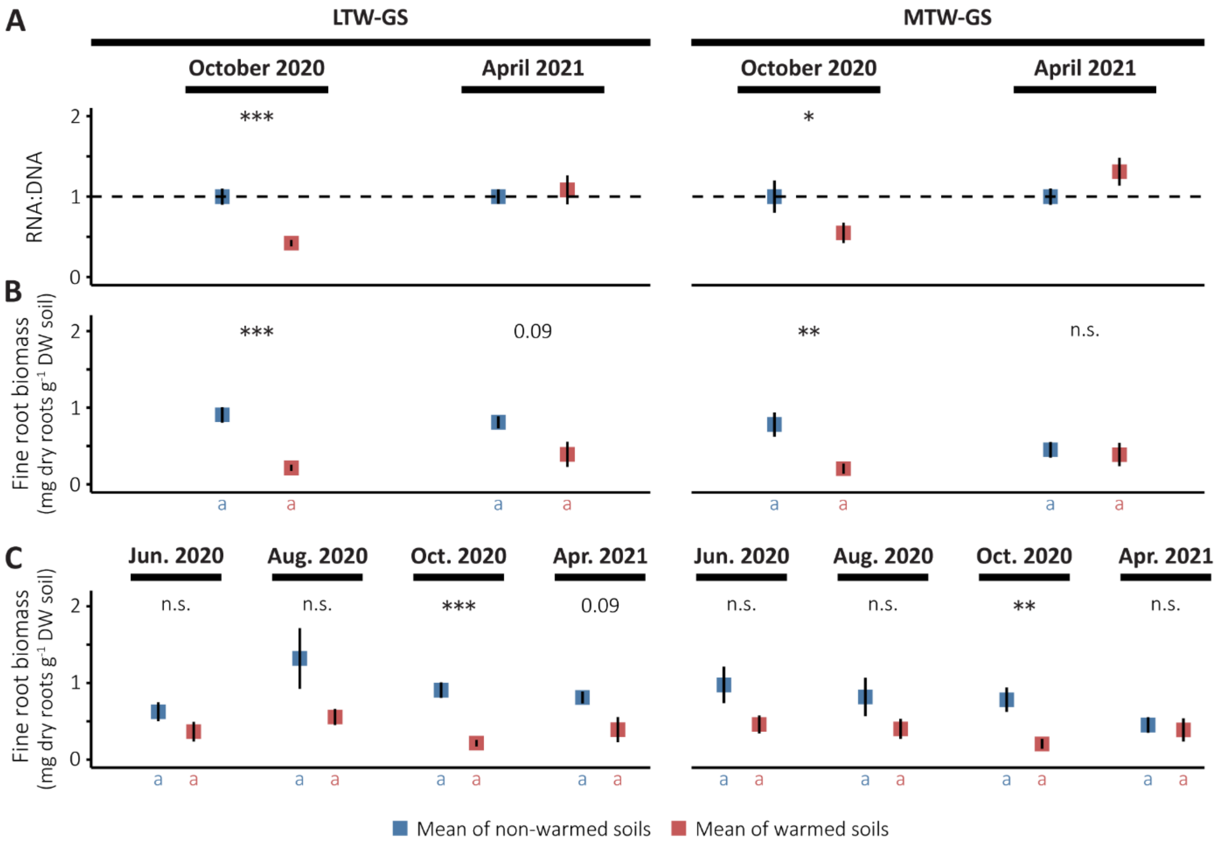

Supplementary Figure S10. RNA:DNA ratios and fine root biomass. (**A**) Mean relative RNA:DNA ratios per site (LTW-GS, MTW-GS) and sampled month (with available fine root biomass data); calculated by setting the mean of non-warmed control soils of each timepoint to one (dashed line). Values above the dashed line indicate an increase in cellular ribosome contents, whereas values below indicate a decrease. Hypothesising that RNA:DNA ratios are lower in warmed soils, one-sided t-tests were performed to test for significant differences; * = $p < 0.05$, *** = $p < 0.001$; see **Supplementary Table S6** for details and exact *p* values. (**B**) Fine root biomass (mg dry weight roots per g dry weight soil) obtained from a second soil core taken the same day as the samples in (**A**). Two-sided t-tests were performed to test for significant differences between non-warmed and warmed soils; ** = $p < 0.01$, *** = $p < 0.001$. (**C**) Fine root biomass (mg dry weight roots per g dry weight soil) obtained from a second soil core taken the same day as the samples in (**A**) (Oct. 2020 and Apr. 2021; i.e., same data as depicted in (**B**)) and two earlier sampling campaigns in June (Jun.) and August (Aug.) 2020. Two-sided t-tests were performed to test for significant differences between non-warmed and warmed soils; ** = $p < 0.01$, *** = $p < 0.001$. Coloured letter code in (**B**) and (**C**) indicate that significant differences in fine root biomass were neither found between the samples taken from the non-warmed plots (blue letters) nor between the samples taken from the warmed plots (red letters). See **Supplementary Table S14** for details and exact *p* values of the two-sided t-tests (**B**) and the pairwise t-tests (**C**). Error bars represent standard error of the mean. Abbreviations: MTW-GS and LTW-GS, medium- and long-term warmed grassland sites; DW, dry weight.
